# Role of Ensemble Deep Learning for Brain Tumor Classification in Multiple Magnetic Resonance Imaging Sequence Data

**DOI:** 10.3390/diagnostics13030481

**Published:** 2023-01-28

**Authors:** Gopal S. Tandel, Ashish Tiwari, Omprakash G. Kakde, Neha Gupta, Luca Saba, Jasjit S. Suri

**Affiliations:** 1 School of Computer Science and Engineering, VIT Bhopal University, Sehore 466114, India; 2 Department of Computer Science and Engineering, Visvesvaraya National Institute of Technology, Nagpur 440010, India; 3 Indian Institute of Information Technology, Nagpur 441108, India; 4 IT Department, Bharati Vidyapeeth’s College of Engineering, New Delhi 110063, India; 5 Department of Radiology, University of Cagliari, 09124 Cagliari, Italy; 6 Stroke Diagnosis and Monitoring Division, AtheroPoint™, Roseville, CA 95661, USA

**Keywords:** magnetic resonance imaging, deep learning, transfer learning, classification, brain tumor, computer-aided diagnosis

## Abstract

The biopsy is a gold standard method for tumor grading. However, due to its invasive nature, it has sometimes proved fatal for brain tumor patients. As a result, a non-invasive computer-aided diagnosis (CAD) tool is required. Recently, many magnetic resonance imaging (MRI)-based CAD tools have been proposed for brain tumor grading. The MRI has several sequences, which can express tumor structure in different ways. However, a suitable MRI sequence for brain tumor classification is not yet known. The most common brain tumor is ‘glioma’, which is the most fatal form. Therefore, in the proposed study, to maximize the classification ability between low-grade versus high-grade glioma, three datasets were designed comprising three MRI sequences: T1-Weighted (T1W), T2-weighted (T2W), and fluid-attenuated inversion recovery (FLAIR). Further, five well-established convolutional neural networks, AlexNet, VGG16, ResNet18, GoogleNet, and ResNet50 were adopted for tumor classification. An ensemble algorithm was proposed using the majority vote of above five deep learning (DL) models to produce more consistent and improved results than any individual model. Five-fold cross validation (K5-CV) protocol was adopted for training and testing. For the proposed ensembled classifier with K5-CV, the highest test accuracies of 98.88 ± 0.63%, 97.98 ± 0.86%, and 94.75 ± 0.61% were achieved for FLAIR, T2W, and T1W-MRI data, respectively. FLAIR-MRI data was found to be most significant for brain tumor classification, where it showed a 4.17% and 0.91% improvement in accuracy against the T1W-MRI and T2W-MRI sequence data, respectively. The proposed ensembled algorithm (MajVot) showed significant improvements in the average accuracy of three datasets of 3.60%, 2.84%, 1.64%, 4.27%, and 1.14%, respectively, against AlexNet, VGG16, ResNet18, GoogleNet, and ResNet50.

## 1. Introduction

Brain or central nervous system cancer is the tenth most prevalent cause of death globally for both men and women, according to the World Health Organization (WHO) [1]. Although brain tumors are not the primary cause of death, according to the cancer statistics report, the main concern is that all other types of cancer can form brain tumors at the metastasis stage and the same was reported in 40% of the cases [1]. Since the year 2000, to spread awareness about brain tumors and to educate people, 8 June is celebrated as world brain tumor day. In the human body, when aberrant cells start to develop abnormally and begin to affect the brain or spinal cord, this condition is known as a brain tumor. The WHO divided brain tumors into four categories ranging from low to high (I, II, III, and IV) based on molecular characteristics and histology [2,3]. At the advanced stage, the life span of a brain cancer patient is very short [4,5]. Therefore, a precise and early tumor diagnosis can aid in selecting the best course of action for treatment which in turn will help in saving millions of lives. The primary indicators of brain tumors are neurologic examination and imaging modalities such as magnetic resonance imaging (MRI) and computerized tomography (CT) [5,6]. Furthermore, biopsy and biomarker tests are advanced methods of tumor grading.

Unlike CT imaging, MRI is a radiation-free method that can produce high-quality images of the inside of the body, which doctors can utilize to determine the location and surgical plan [7,8]. Additionally, the patients are assessed before and after the treatment and disease progression can be monitored. MRI is available with various sequences, such as fluid-attenuated inversion recovery (FLAIR), T1-weighted MRI (T1w), T1-weighted contrast enhancement (T1Wc), T2-weighted (T2W), and T2-contrast with (T2Wc). Due to differences in the physical method of image capturing, tissue structures appear differently in each sequence image [7,9,10]. Therefore, we anticipate that each MRI sequence will yield different results in brain tumor classification. Based on the earlier research, we analyzed that a suitable MRI sequence for brain tumor classification has yet to be discovered. Thus, the main objective of this research is to find an appropriate MRI sequence for brain tumor classification. The biopsy is the industry-standard method for grading tumors by examining the color, size, shape, and distribution of tissue in a visible tumor sample [11] [12,13]. The complete tumor grading method is a difficult and time-consuming process that involves (i) a physical or neurological examination, (ii) the detection of the tumor, (iii) an evaluation of its size, form, and position within the body, (iv) a surgical resection for a biopsy, (v) a tissue analysis, and (vi) finally, decision-making for tumor grading. The gold standard for estimating the stage of a tumor is a biopsy [11,14,15]. However, tissue analysis in the biopsy is time-consuming, prone to the risk of error, and subject to inter-observer variance. Therefore, a quick, automated, non-invasive computer-aided diagnosis (CAD) tool is needed for brain tumor patients, since the number of cancer patients rises over time [11,16,17,18,19].

With the invention of efficient artificial intelligence (AI) methods, various CAD tools were proposed for multiple medical applications [20,21,22,23]. Mainly, the two major branches are constituted of efficient AI algorithms, namely, (1) machine learning (ML) [24,25,26,27] and (2) deep learning (DL) [28,29,30]. The conventional ML techniques are extensively used in many CAD tools for applications such as coronary artery disease [31,32], diabetes [33,34], and classification of skin [35,36], thyroid [37,38], liver [39,40,41], ovarian [42,43,44], and prostate cancers [45]. The major challenge of ML-based algorithms is feature selection or feature enrichment [46]. There could be endless features possible for medical data, and within that context, suitable feature selection is a complicated task [35,47]

**Figure 1:**
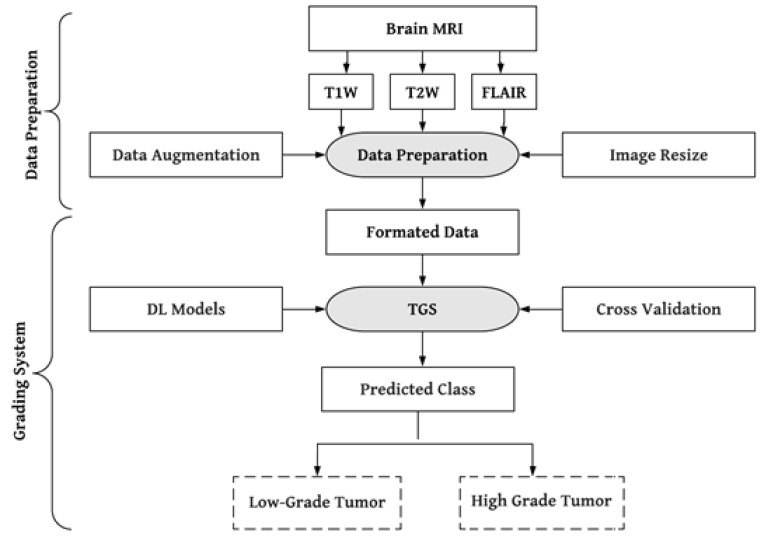
A global picture of brain tumor grading system. T1W: T1-weighted, T2W: T2-weighted, TGS: tumor grading system.

Convolutional neural networks (CNN), which automatically extract the most appropriate features from images, have significantly benefited from deep learning (DL) solutions by adding a new dimension to the feature extraction process [48,49,50,51,52]. The use of DL algorithms allows the extraction of highly minute details that are not even visible to the human eye [26]. As a result, DL techniques are widely employed in medical image analysis for tasks including image registration [53], segmentation [54,55,56,57], and classification [58,59,60,61,62]. The most frequent type of brain tumor in people is glioma. We have consequently suggested a DL-based effective brain tumor grading method to categorize gliomas into low-grade (LGG) and high-grade (HGG). Figure 1 presents the system’s overview. The literature review section addresses the issues found in earlier investigations.

The overview of the whole paper is as follows. The introduction is in Section 1. Materials and methods are covered in Section 2. The results are covered in Section 3. The discussion of the study is given in Section 4, and the conclusion is given in Section 5.

In the early years, the engineering and medical domains were segregated, but with the advancement in the AI vertical of engineering, it is possible to unearth many mysteries of the medical field. Therefore, AI is used in many medical or healthcare applications such as automatic disease, prognosis, diagnosis, treatment assessment, and planning [2,22,23]. DL offers superior performance as compared to ML with effortless feature extraction and efficient classification [63,64]. Among the DL techniques, the CNN model is quite popular and is applied in many applications such as cancer diagnosis [2,65], ischemic lesion detection and segmentation [66], histopathology image analysis [12,13,67,68,69], etc.

CNN models are widely employed in applications of computer vision. Its power was revealed after the ImageNet competition [70,71], where various well-known CNN models were proposed and benchmarked on an ImageNet dataset. This dataset consists of millions of real-world images in more than a thousand classes. The concept of CNN models is not new, but was rather invented two to three decades ago. However, the popularity of CNNs proliferated due to the rise of graphics processing units (GPUs). The computational power of computers has increased manifold due to GPUs, which in turn has revolutionized AI-based applications using deep learning. Here, we summarize some of the best AI-based existing works in brain tumor classification (BTC).

For tumor detection from an MRI image, a modified InceptionResNetV2 pre-trained model is employed by Gupta et al. [72]. Three tumor classes were designed, including glioma, meningioma, and pituitary cancer. Due to the dataset’s limited size, they used Cyclic Generic Adversarial Networks to increase the dataset size. A combined model with InceptionResNetV2 and Random Forest Tree was proposed for classification. The model achieved 99% and 98% accuracy for the suggested tumor classification and detection models, respectively. Haq E et al. [73] proposed a hybrid approach for brain tumor segmentation and classification by integrating DL and ML models. The tumor region’s image space was used to generate the feature map. A faster region-based CNN was also created for tumor localization, followed by a redesign of the region proposal network. Furthermore, CNN and ML are combined in such a way that they can improve the accuracy of the segmentation and classification processes. The suggested technique attained the maximum classification accuracy of 98.3% between gliomas, meningiomas, and pituitary tumors. Srinivas et al. [74] used transfer learning to test three CNNs for brain tumor classification: VGG16, ResNet50, and Inception-v3. The VGG16 has the best accuracy of 96% in classifying tumors as benign or malignant. Almalki et al. [75] classified tumors using a linear machine learning classifiers (MLCs) model and a DL model. Transfer learning method is utilized to extract MRI features from a designed CNN. The proposed CNN with several layers (19, 22, and 25) is used to train the multiple MLCs in transfer learning to extracting deep features. The accuracy of the CNN-SVM fused model was higher than that of previous MLC models. The fused model provided the highest accuracy (98%).

Kibriya et al. [76] suggested a new deep feature fusion-based multiclass brain tumor classification framework. A min-max normalization technique with data augmentation is used as a preprocessing step. Deep CNN features were extracted from transfer learning architectures such as AlexNet, GoogleNet, and ResNet18 and fused to create a single feature vector. SVM and KNN models are used as a classifier on this feature vector. On a 15,320 MR-image dataset, the suggested framework is trained and evaluated. According to the results of the investigation, the fused feature vector outperforms the individual vectors. Furthermore, the proposed technique outperformed the current systems, achieving 99.7% accuracy. Gurunathan et al. [77] suggested a CNN Deep net classifier for detecting brain tumors and classifying them into low and high grades. The ROI is segmented using global thresholding and an area morphological function. The suggested model extracts the features from the augmented image internally. For classification and segmentation, the suggested method is totally automated. Furthermore, based on its feature properties, the suggested technique claims segmentation and classification accuracy of 99.4% and 99.5%, respectively.

Alis et al. [78] presented research utilizing ANN for glioma classification between LGG and HGG. A total of 181 patients participated in the study, of whom 97 were HGG and 84 were LGG. They used the MRI data in contrast-enhanced T1W, T2W, and FLAIR sequences and extracted the ROIs manually. They used handcrafted features such as higher-order texture features and histogram parameters for the classification. The T2W-FLAIR dataset’s area under the receiver operating characteristic curve (AUC) for a test cohort of 60 patients was 0.87, and the contrast-enhanced T1W dataset’s AUC was 0.86. The highest degree of diagnosis accuracy had an AUC of 0.92 and was 88.3%. In a CNN-based study, Khavaldeh et al. [79] classified MRI scans into healthy, LGG, and HGG groups. The authors used 130 patients’ publicly available REMBRANDT brain tumor data. They merged the grade 2 oligodendroglioma and grade 2 astrocytoma to form the LGG class. The HGG class of tumors included astrocytoma (grade 3), oligodendroglioma (grade 3), and glioblastoma-multiforme (GBM) (grade 4). In the third category, ‘healthy’, they included healthy MRIs. The labeling of the data samples was performed at the image level rather than at the pixel level. The proposed CNN produced the most significant classification accuracy of 91.61%. For brain tumor grading, Anaraki et al. [80] presented a genetic algorithm (GA)-based CNN framework for grading brain tumors. In this method, an optimal CNN model was generated by employing a trial-and-error process for parameter selection. The proposed model achieved a classification accuracy of 94.2% for gliomas, meningiomas, and pituitary cancers.

Yang et al. [69] presented a brain tumor classification method using the TCIA dataset. Data were classified between LGG and HGG classes using an ROI-based segmented method. Two well-established models: AlexNet and GoogleNet were used in the study. The study compared two methods of training; in the first method, the models were trained from scratch, while in the second method, the models were trained with a transfer learning technique. The transfer learning method has shown better performance than training the model from scratch. GoogleNet achieved the highest accuracy of 94.5% in the transfer learning paradigm. Swati et al. [81] used a pre-trained deep CNN model to solve the tumor classification problem. This approach proposed a block-wise fine-tuning technique based on transfer learning. The proposed method is flexible as it does not depend on handcrafted characteristics. They achieved 94.82% accuracy wih minimal preprocessing on the contrast-enhanced-magnetic-resonance-image (CE-MRI) dataset.

Badza et al. [82] proposed their own CNN architecture for three types of brain tumor classification. The proposed model is more straightforward than existing pre-trained models. They used T1W-MRI data for the training and testing with 10-fold cross-validation. The highest classification accuracy of 96.56% was achieved through the proposed model for three-class brain tumor data. An automatic content-based image retrieval system was introduced for the feature selection of brain tumors using T1-weighted contrast-enhanced MRI [83]. The authors used the DL-based feature extraction method in the TL framework and adopted a closed-form metric learning method to measure the similarity between the query image and database images. The five-fold cross-validation was adopted with an average precision of 96.13% on 3064 images. Segmentation and classification are essential aspects of the brain tumor grading system. The segmentation is challenging due to the varying sizes of images in massive datasets. Hence, an optimized method, ‘Dolphin Echolocation-based Sine Cosine Algorithm’, was suggested by [84] based on CNN. They performed the segmentation via a fuzzy deformable fusion model with the proposed algorithm and used statistical features, such as mean, variance, and skewness, for classification using CNN. The proposed method has shown a maximum accuracy of 96.3% during classification.

Another study [85] proposed a DL-based method for brain tumor segmentation and classification. In the first phase, the texture features were extracted by an inception-based V3 pre-trained CNN model. Later on, the feature vector was optimized using the particle swarm optimization method. The segmentation method was validated on BRATS2017 and BRATS2018 datasets; a dice score of 83.73% for the core tumor, 93.7% for the whole tumor, and 79.94% for the entire enhanced tumor was achieved. Similarly, on the BRATS2018 dataset, a dice score of 88.34% (core), 91.2% (whole), and 81.84% (enhanced) was attained. In the classification phase, an average accuracy of 92% was achieved on the BRATS 2013, 2014, 2017, and 2018 datasets. Similarly, in a study [86], the authors compared CNN classification performance on three MRI datasets: cropped, uncropped, and segmented lesion images. During the experiments, 98.93% classification accuracy was seen in the cropped lesions image dataset, and 99% accuracy was observed in the uncropped lesions image dataset. Further, with segmented lesion image datasets, they attained 97.62% accuracy. Another study [63] proposed a multiclass framework for brain tumor classification. The authors designed five multiclass datasets, such as two-class, three-class, four-class, five-class, and six-class, for inter- or intra-tumor grade classification. MRI images were partially segmented in the datasets. The CNN model (AlexNet) was used in the transfer learning paradigm and benchmarked its performance against six different machine learning models: decision tree, linear discrimination, naive Bayes, support vector machine, K-nearest neighbor, and ensemble. The CNN outperformed all other ML models in the classification performance. They adopted three kinds of cross-validation protocols (K2, K5, and K10) during the training, and their mean accuracies for two-, three-, four-, five-, and six-class datasets were 100, 95.97, 96.65, 87.14, and 93.74%, respectively, for *p* < 0.0001.

After analyzing the above studies, we identified some challenges such as; (1) earlier studies used MRI data in various MRI sequences such as T1W, T2W, FLAIR, and so on. We analyzed the three most frequent MRI sequences, T1W, T2W, and FLAIR data, to determine an appropriate MRI sequence that could improve the performance of brain tumor classification. (2) In previous similar studies, several researchers worked on many models. The models showed uneven performance on different datasets, in which the performance of the best model was taken into account. We used the opinion of other models and ensembled them in this study to generate consistent and enhanced performance. (3) Over-fitting is a common problem when deep learning models are trained with limited medical data. Overfitting occurs when a model performs well on known data but fails to recognize unseen data. Unfortunately, medical brain data are scarce. Many initiatives were undertaken to address this issue, including transfer learning, dropout connection, data augmentation, and five-fold cross-validation of data. Further, we have included the comparative table of earlier proposed work in Table A1 of Appendix A.

### The Significant Findings of the Proposed Work Are as Follows

- To develop an efficient computer-aided diagnosis tool for brain tumor grading.
- Finding a suitable MRI sequence for the brain tumor classification.
- Proposed ensemble algorithm based on majority voting.

## 2. Materials and Methods

Brain tumor data known as “Molecular brain tumor data (REMBRANDT)” was gathered from the public data repository Cancer Imaging Archive (TCIA) [87,88]. The dataset was originally developed by Thomas Jefferson University (Philadelphia, PA, USA) and Henry Ford Hospitals (Detroit, MI, USA). The dataset contains MRI data from 130 patients, divided into three brain tumor types, astrocytoma (AST), oligodendroglioma (OLI), and glioblastoma-multiforme (GBM). Tumor type, AST, and OLI were available as grade-2 (g2) and grade-3 (g3). At the same time, GBM was available in grade-4 (g4). As per the available fact sheets, the ground truth of 15 patients was not available, and the data of 27 patients needed to be labeled appropriately. In the dataset, a total of 88 patients with brain tumor types of AST (47), OLI (18), and GBM (23) had valid ground truth. Tumor-type AST included 30 patients with g2 and 17 patients with g3, while tumor-type OLI included 11 patients with g2, and 7 patients with g3, and tumor-type GBM included only 23 patients with g4.

### 2.1. Data Preparation

**Figure 2:**
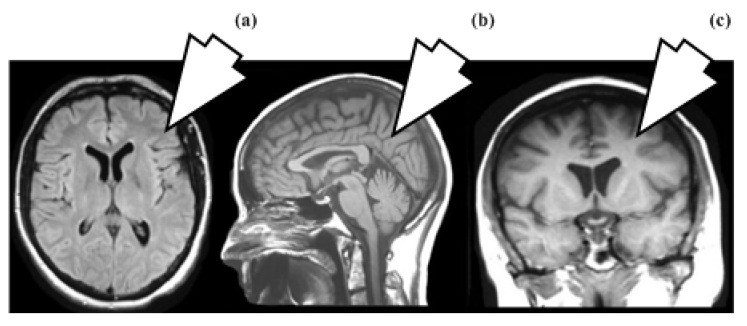
(**a**) Axial, (**b**) Sagittal, (**c**) Coronal views.

No preprocessing was employed because image-enhancing processes could change the original tumor characteristics. Segmenting tumor areas is a complex and time-consuming process. We therefore took the entire MRI slice as a sample following the idea that “CNN can extract relevant features from the image”. This idea avoids not just unneeded computing work but also segmentation overhead. Typically, MRI slices are captured in axial, sagittal, and coronal views, as shown in Figure 2. In the proposed study, whole brain 2D MRI slices are taken in axial view.

**Figure 3:**
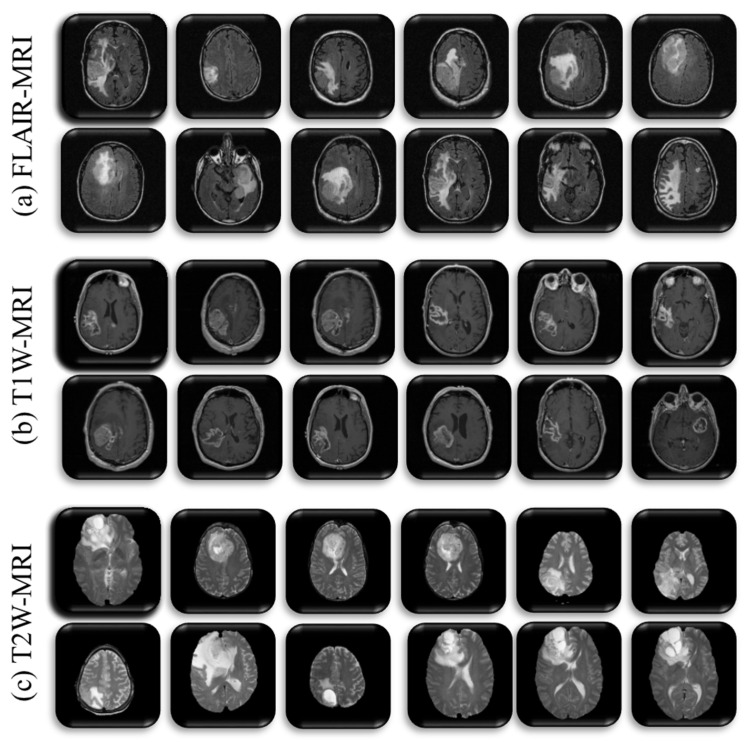
Sample images of three MRI sequences dataset, (**a**) T1W, (**b**) T2W, (**c**) FLAIR.

**Figure A1:**
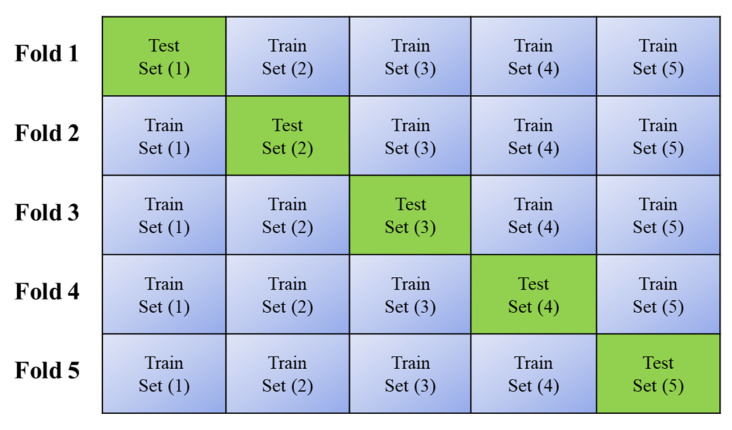
An idea of five-fold cross-validation.

This study aims to classify the most prevalent gliomas in the brain into low-grade and high-grade tumors. For brain tumors, we need to know the best possible MRI sequence to classify LGG against HGG with the most significant degree of accuracy. Therefore, the three most popular MRI sequences of all patients, T1W, T2W, and FLAIR, were taken and we created their three datasets. In the context of some of the earlier studies [69,89,90], the LGG and HGG classes were constituted. The g2 patients of tumor type AST and OLI were included in LGG class, whereas g3 patients of AST and OLI types, and g4 patients of GBM were included in HGG class. Forty-four patients were present in the LGG class, and 68 patients were included in the HGG category. Sample details of each class of three MRI-sequence datasets are summarized in Table 1 and sample distribution of five-fold cross-validation is depicted by Figure A1 and Table A2 in Appendix A. Some representative samples of the above three datasets are depicted in Figure 3.

### 2.2. Preprocessing

The data augmentation method was adopted in the proposed study to avoid overfitting. The brain slices were rotated randomly between (−30 to 30) degree angles and we scaled the images randomly between factors (0.9 to 1.1). Further, we resized the images as per the input requirements of the CNN models. Furthermore, we adopted a five-fold cross-validation protocol, where five rounds of training and testing were performed on randomly selected data with 80% training and 20% test samples.

### 2.3. Clinical Relevance of MRI Sequence

MRI is a medical imaging technique used in radiology to form pictures of the anatomy and the physiological processes of the body. This technique uses magnetic fields and radio waves to create images by distinguishing between the nuclear magnetic properties of different tissues. The human body consists primarily of water and fat molecules that can release massive amounts of hydrogen (H+) as a source of protons. Tissues are made of protons, and they behave like magnet bars and have positive and negative poles. This property of protons is used to interact with specific radio waves of MRI scanners. Therefore, MRI maps the amount of water and fat in the body. The contrast and illumination in the image are defined by the protons’ density. The resulting images look lighter if the protons are densely populated in tissue and appear darker in much less populated tissue. Some other factors, such as the relaxation time of protons, are included for defining the MRI. The relaxation process includes T1 relaxation time and T2 relaxation time. The T1 relaxation time is the reorientation time with the magnetic field through the 63% proton after the radio wave pulse has stopped. Similarly, the time to stop spinning 37% of the protons after closing the radio wave pulse is known as the T2 relaxation time. Therefore, MRI differentiates the tissue based on the release of energy by the proton after the radio wave pulse has stopped. MRI constructs a map based on these tissue differences with the help of a computer that connects to the scanner, collects all information through mathematical formulas, and produces a 2D or 3D image.

Different types of MRI sequences or protocols can be created through unique settings of radio-frequency pulses and gradients and can be used for differential applications. T1W, T2W, FLAIR, and proton density-weighted images are the most popular MRI sequences. To comprehend these protocols, a few additional words are necessary, such as repetition time (TR) and time to echo (TE). The time interval between consecutive pulse sequences applied to uniform slices is known as the TR. Similarly, TE measures the interval between the radio wave’s pulse onset and the moment its resonant signal is received. The combination of these TR and TE times defines the above MRI sequences. Due to the heterogeneous nature of the tumor, the single MRI protocol is insufficient to express the tumor structure [7]. Similarly, each MRI protocol has a specific clinical relevance [91,92]. This study adopts three significant MRI sequences’ data to find an appropriate MRI sequence for automated brain tumor detection and grading. The clinical relevance of each MRI sequence is as follows.

Short TR and short TE signals are used to create T1W pictures. The T1 characteristics of the tissue, which define its primary clinical differences, define the contrast and brightness in this image. The pre-contrast T1W evaluates the tissue structure by highlighting melanin, mineralization, and blood components with high intensity. Meanwhile, using a contrast agent (Gadolinium-DTPA), contrast-enhanced T1W images highlight proliferative tumor regions due to the accumulation of contrast agents around the tumor. Longer TR and TE signals are used to create T2W sequence images. In contrast to T1W images, it has the opposite clinical distinctions and evaluates how quickly the tissue loses its magnetization. High grey and white matter contrast are produced because the free water signal is suppressed. Consequently, the edema region from the cerebrospinal fluid (CSF) is separated, and the tumor region appears bright. Low-intensity hemorrhage is usually seen with tumor vascularity, calcification, and when it is radiation-induced. CSF, which is darker in T1W images, can distinguish T1W and T2W images from one another. However, in T2W images, it appears brighter. However, TR and TE are significantly longer than T1W and T2W. FLAIR and T2W are roughly comparable. This protocol is designed to suppress the signal of water contents or fluids, including CSF, so CSF appears dark. It increases lesion conspicuity, producing enhanced visualization of vasogenic edema, gliosis, and infiltration of tumors near the cortex and ventricles [92]. Likewise, it is helpful for imaging in cases of meningitis, subarachnoid hemorrhage, multiple sclerosis plaques, and lacunar infarctions. PD-weighted images are typically used to visualize disorders of the joints and brain. As the name says, this sequence measures the proton density per volume. The resulting high-density proton tissue produces a high-intensity signal; conversely, a low proton density in the tissue creates a low-intensity signal. The clinical relevance of PD sequence is as follows: joint injuries, gray and white matter contrast brain image, CSF, and tissue contrast in undergoing the pathological process.

### 2.4. Methodology

During the last decade, most researchers turned towards deep learning frameworks to handle image classification problems using various deep neural networks. At the same time, CNN is the most popular approach adopted in various computer vision applications, which has proved successful in terms of efficiency and accuracy [76,93]. Therefore, CNNs are established as a reliable class of models for various pattern recognition problems, including face detection and recognition, object detection and recognition, picture segmentation, image retrieval, and classification. The success of CNN in terms of increased performance as a classifier prompted more scientists to conduct their studies using the CNN technique. As a result, a variety of CNN models have been proposed recently. CNN’s optimization techniques are broadly classified into two categories, model ensembling and multilayer architecture design with appropriate parameter selections. Numerous effective CNN models have been presented, and a significant amount of study has already been conducted on creating multilayer architectures with suitable parameter values. We investigated the model ensembling strategy for enhancing CNN performance in our work. Five well-known CNN models, including AlexNet (8-layer), VGG16 (16-layer), ResNet 18 (18-layer), GoogleNet (22-layer), and ResNet 50 (50-layer), are combined for this purpose. All models are trained in transfer learning mode. This helps prevent the overfitting issue when training models with limited medical data.

**Figure 4:**
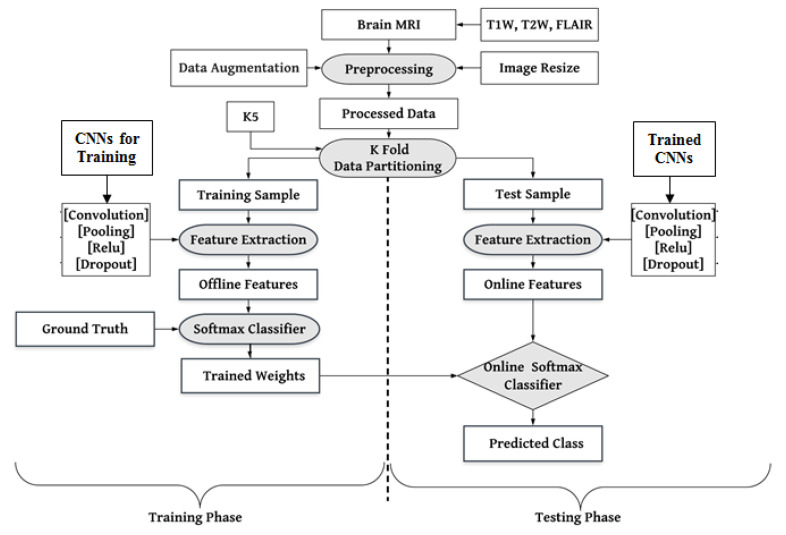
Local system architecture for training and testing of pre-trained CNNs: K5-five-fold.

As mentioned above, the architectures of the CNNs were modified as per desired labeled data. We removed the CNN architecture’s topmost fully connected (FC) layer and included new FC layers for our desired binary labels (LGG and HGG). The working mechanism of the proposed CAD tool is discussed in Figure 4. Three MRI sequences’ (T1W, T2W, and FLAIR) data have been included. The researcher proposed many initiatives to resolve the overfitting issue, such as resizing and augmenting datasets in the preprocessing step. The training and testing of the dataset followed a five-fold (K5) cross-validation strategy, wherein 80% of training and 20% of test samples were divided into five random folds (sets). Based on the idea of transfer learning, all the models were initialized to initial weights (knowledge) of raw data before being fine-tuned on brain tumor MRI data. The model architectures, their comparison, and the suggested ensemble algorithm for performance optimization are covered in depth below.

### 2.5. Transfer Learning

**Figure 5:**
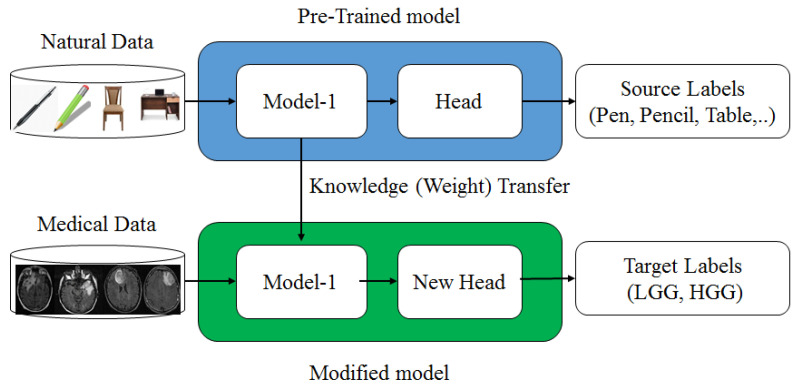
Transfer learning mechanism.

In the absence of a large amount of labeled data, CNN is quite difficult to train from scratch and requires a great deal of expertise to ensure sufficient convergence. Therefore, fine-tuning a CNN from pre-trained networks is an alternative to using TL. Prior knowledge of the TL methods can be utilized for new tasks where training data is limited. This method was successfully used in many medical domains where the available medical dataset is limited [50,69,94]. However, most of the available pre-trained networks (CNNs) are trained on natural images. In contrast, there is a significant difference between natural and medical images. So, a questioning session is generated so that the knowledge of natural images can be transferred to medical images. For three reasons, TL is an essential and effective method for deep learning models. (1) TL has attained notable successes in the medical domain where medical data is limited for many applications, such as in Tandel et al. [63], Paul et al. [95], Sultan et al. [96], and Sajjad et al. [97], and TL was also successfully utilized for glioma grading, epileptic electroencephalogram recognition [98], brain hemorrhage [99], lung cancer [100], prostate cancer [101], etc. (2) ML models trained on handcraft features cannot address TL because the learning process is limited to selected features only. (3) The TL can speed up the learning process and reduce the risk of overfitting during the training. The complete procedure of TL is depicted in Figure 5, where the model pre-trained on raw data transfers its weights to the revised model, which will be trained on medical data for new labels.

### 2.6. Pre-Trained Convolution Neural Network

This study applied the transfer learning paradigm to five well-known pre-trained CNN models: AlexNet, VGG16, ResNet18, GoogleNet, and ResNet50. These models were previously trained on the ImageNet dataset. The architecture discussion of each model is given below.

#### 2.6.1. AlexNet

Alex Krzyzewski first suggested AlexNet in the Large-Scale Visual Recognition Challenge (ILSVRC) in 2012 [102], which won first place in this contest. The top-5 error was 15.3%, which was less than 10.8% of other states of the arts of that time. The shallow network known as AlexNet was originally trained on two GPU machines; however, these days, only a single GPU is sufficient. AlexNet is an eight-layer deep network and consists of five convolution layers (Conv), followed by three fully connected (FC) layers. These layers with three filter sizes (11 × 11, 5 × 5, and 3 × 3), max pooling, dropout, and data augmentation operation were included. In the same model, the traditional sigmoid function *SF*(*x*) (Equation (1)) was replaced by a rectified linear unit (Re*Lu*) (Equation (2)) as an activation function because the sigmoid function was suffering from a vanishing gradient problem, where the learning process stops when the gradient falls to zero.

(1) $$SF(x)=\frac{1}{1+\exp(-x)}$$

(2) ReLu(x)=Max(0,x)

#### 2.6.2. VGGNet

This model was initially developed in the Visual Geometry Group (VGG) Lab of Oxford University in 2014. The VGG model was designed by Karen Simonyan and Andrew Zisserman and won the first and second positions of the ILSVRC-2014 challenge [103] with top-5 test accuracy of 92.7%. This model is available in many layering versions (16 and 19 layers). Here we have used the VGG16 model, which consists of 13 Conv layers and 3 FC layers. The very small (3 × 3) convolution filters are used throughout all the Conv layers with a stride size of 1 and the same padding. Further, (2 × 2) filters are used for pooling with a stride of size 2. The default input size of the image of the VGG16 model was 224 × 224.

#### 2.6.3. GoogleNet

This model was proposed by the research group of Google in 2014 and a paper was published with the title “Going Deeper with Convolutions” [77]. It secured the first position in the ILSRVRC 2014 competition with a top-5 error rate of 6.67% in the classification task. The architecture of GoogleNet consists of 22 layers, and this design was inspired by LetNet [84]. The designer’s goal was to use very small convolution filters (1 × 1) to limit the number of intermediate parameters. Consequently, it reduced the 60 million (AlexNet) parameters to 4 million. This architecture was somewhat different from earlier models; some of the major highlights of the model are as follows. (1) 1 × 1 filter limits the number of parameters (weights and biases). (2) At the network’s end, the feature map of (7 × 7) was averaged to (1 × 1) using the global average pooling technique. The top-1 accuracy increases by 0.6% as a result, while the number of trainable parameters drops to 0. (3) A fixed Convolution (1 × 1, 3 × 3, and 5 × 5) and 3 × 3 max-pooling were carried out concurrently in the inception module. The main goal of this module is to handle objects at various scales more effectively. (4) In the middle of the training architecture, certain intermediate classifier branches, such as the Auxiliary classifier, are introduced. This mechanism provides regularization and helps to avoid the vanishing gradient issue [104,105].

#### 2.6.4. Residual Net

The Residual Net (ResNet), developed by Kaiming and his team for Microsoft research, was first presented at ILSVRC 2015 [79]. In the same competition, this mode won the classification job with a 3.57 percent error on the ImageNet test set. Lowering the cost of layer depth in computation time and speed is a significant advantage of this architecture. ResNet topologies were put forth in layering variations, such as versions 18, 50, 101, etc. In this study, we employed the Residual Net deep architecture with ResNet18, which has 18 layers, and ResNet50, which has 50 layers. Table 2 compares some of the unique features of the CNNs as mentioned above [106].

### 2.7. Majority Voting Algorithm (Algorithm 1)

**Figure 6:**
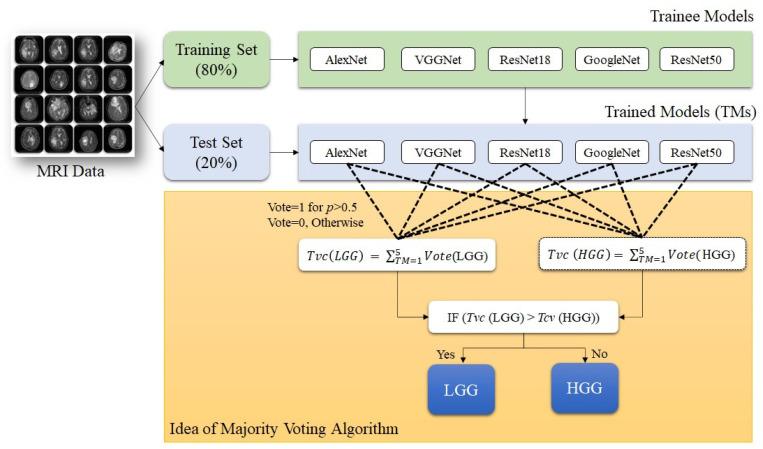
Pictorial representation of MajVot algorithm.

As discussed above, in earlier research articles, CNN-based classification of brain tumor images was emphasized as the single highest-performing model. As a result, the potential of the rest of the models needed to be utilized in a multimodal environment. In this study, we have used five models, and the prospect of all the models is utilized using an ensemble algorithm for performance optimization for brain tumor classification. The proposed ensemble algorithm is based on the probabilistic prediction for desired labels and the majority voting (MajVot) mechanism of five models. The votes of each class label (LGG and HGG) were calculated using the projected probability of each CNN for each test sample. For example, if Model-X predicts the predicted probability of the LGG label to be greater than 0.5, the value of Model-X’s vote for the label ‘LGG’ will be one; otherwise, it will be zero. Similarly, based on the prediction of each model’s class label, the vote value is derived. Finally, the estimated votes for each class label will be summed. This was the preprocessing step for the MajVot algorithm. It predicts class labels based on the following rule: if the total vote share of label LGG is greater than HGG, then LGG will be selected; otherwise, HGG. This process will be repeated for all the test samples. Ensure that the number of voters (models) is odd to avoid a tie between the two classes. A pseudo-code representation of the MajVot algorithm is given below. A pictorial representation of the algorithm is described in Figure 6.
**Algorithm 1**: Majority Voting.**Input**: n Models or classifiers, training samples with ground truth and test samples.**Output**: Predicted class labels, label probability score, and performance evaluation.**Step 1**. Train all n models on the same training set. **Step 2**. Take a sample from test set and test it through trained model and predict the label in terms of probability score.**Step 3**. Measure a model’s vote for each label by the following rule.            **IF** Probability (label ‘**LGG**’) > 0.5 **THEN**
                  Vote (LGG) = 1 and Vote (HGG) = 0.            **Otherwise**,                   Vote (LGG) = 0 and Vote (HGG) = 1**Step 5.** Repeat step 2 and 3 for all the trained models.**Step 6.** Calculate the total number of votes for each label predicted by all trained models by the following rule.           **IF** (Total Vote ‘**LGG’)** > (Total Vote ‘**HGG’) THEN**
                 Label ‘**LGG’** will be predicted.           **Otherwise,**
                 Label ‘**HGG’** will be predicted.**Step 7.** Repeat **Step 2** to **Step 6** for all the test samples.**Step 8.** Compare predicted labels of each sample to the actual ground truth and create confusion matrix.

## 3. Results 

**Figure A2:**
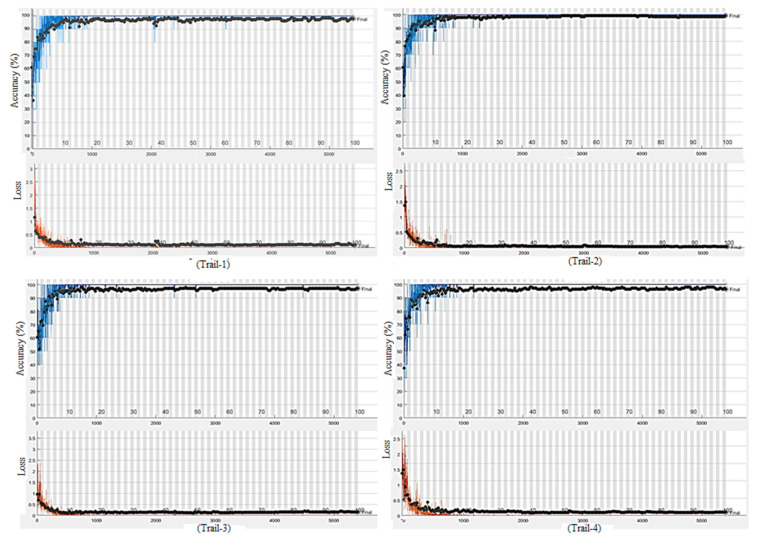
Sample training curve of four rounds of the experiment (ResNet18, FLAIR).

**Figure A3:**
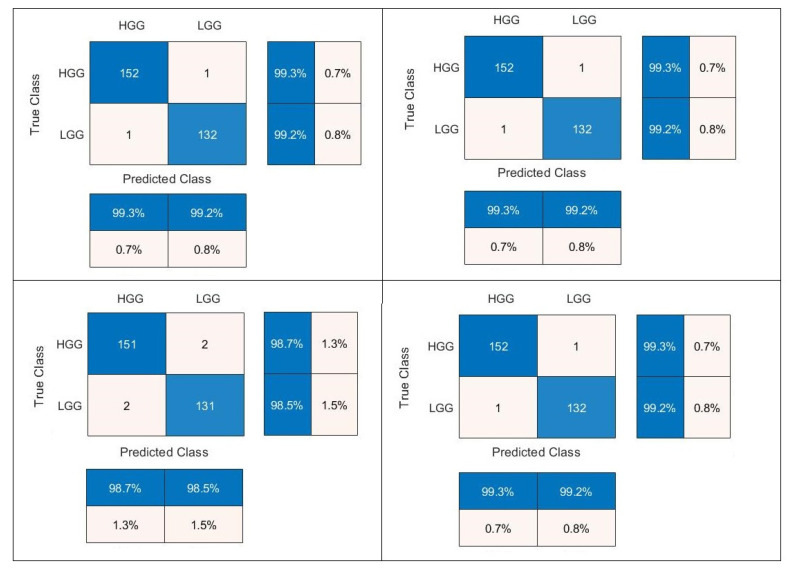
Confusion matrix of four rounds of the FLAIR-MRI dataset using the MajVot algorithm.

**Figure A4:**
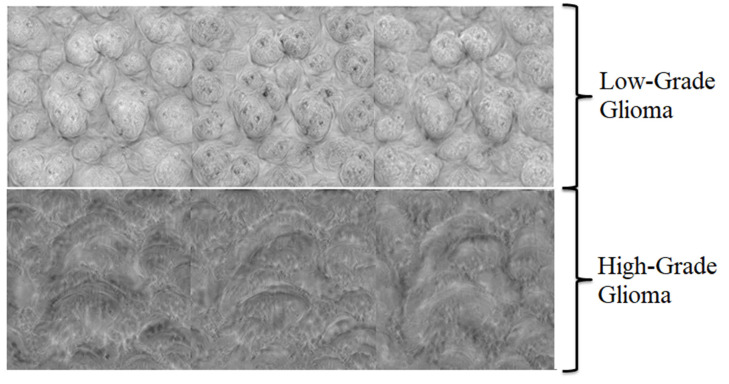
Heatmap diagram of head node for low-grade and high-grade glioma.

Based on the comparison of anticipated labels and actual labels, we assessed the test performance in terms of true positive (*TP*), true negative (*TN*), false positive (*FP*), and false negative (*FN*). Further, the performance parameters such as accuracy (*ACC*), sensitivity (*SEN)*, specificity (*SPC*), positive predictive value (*PPV*), negative predictive value, and area under the curve (AUC) were evaluated from the above-mentioned basic parameters. The mathematical expression of *ACC*, *SEN*, *SPC*, *PPV* and *NPV* are described by Equations (3), (4), (5), (6) and (7), respectively. A total of 18 experiments were designed from six models (AlexNet, VGG16, ResNet18, GoogleNet, ResNet50, and the MajVot algorithm) and three datasets (T1W-MRI, T2W-MRI, and FLAIR-MRI). The total combinations of experiments are described in Table 3. Additionally, a five-fold (K5) cross-validation process was performed, where five rounds of training and testing were conducted with different training (80%) and test (20%) samples. Therefore, we completed 90 rounds of training and testing in the 18 experiments. The entire experiment was performed on a trial version of Matalab2021b software, which is freely available on the official website of Matlab [86]. The simulation was performed on an i5 processor with a 4 GB NVIDIA graphics card. The average training time of each trial was 275 min, and the total training time of all the rounds (90) was approximately 412 h. The initial training parameters of CNNs experiments are given in Table 4. Figure A2, Figure A3 and Figure A4 of Appendix A show sample training curves, confusion matrices, and heatmap diagram of intermediate results.

(3) $$ACC=\frac{\left(TP+TN\right)}{\left(TP+TN+FP+FN\right)}\times 100$$

(4) $$SEN=\frac{\left(TP\right)}{\left(TP+FN\right)}\times 100$$

(5) $$SPC=\frac{\left(FP\right)}{\left(TN+FP\right)}\times 100$$

(6) $$PPV=\frac{\left(TP\right)}{\left(TP+FP\right)}\times 100$$

(7) $$NPV=\frac{\left(TN\right)}{\left(TN+FP\right)}\times 100$$

### 3.1. Performance Evaluation of Dataset

This section describes the classification performance of three MRI sequence datasets using five pre-trained CNNs and the proposed MajVot algorithm. The five-fold cross-validation performance is described by $\partial_{\left(DS_{i,}M_{k}\right)}$ in Equation (8), where *DS_i_* is a dataset number (*i* = 1:3), and *M_k_* is the model number (*k* = 1:6). Further, this variable is a set of six parameters such as accuracy $\left(\partial_{ACC}\right)$, sensitivity $\left(\partial_{SEN}{}_{}\right)$, specificity $\left(\partial_{SPC}\right)$, positive predicted value $\left(\partial_{PPV}\right)$, and negative predicted value $\left(\partial_{NPV}\right)$. Each parameter is the average of five trials of each experiment. Their mathematical expressions are described in Table 5. The performance analysis of the three datasets is as follows.

(8) $$\partial_{\left(DS_{i,}M_{k}\right)}=\left(\partial_{ACC},\partial_{SE}{}_{N},\partial_{SP}{}_{C},\partial_{PPV},\partial_{NPV},\partial_{AUC}\right)$$

#### 3.1.1. T1W-MRI Data Analysis

**Figure 7:**
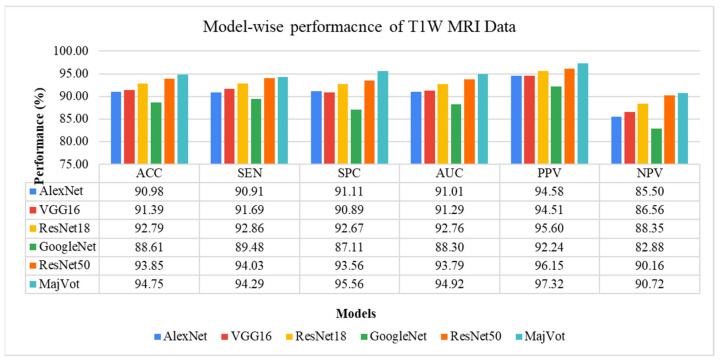
Five-fold test performance of T1W-MRI Data.

**Figure 8:**
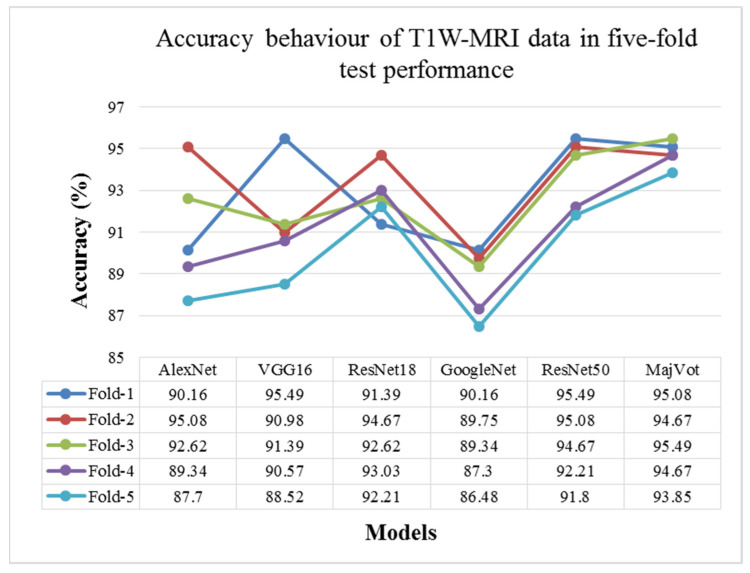
Model-wise accuracy behavior in the five-fold test performance of T1W-MRI data.

In this study, glioma was classified as low-grade (LGG) and high-grade (HGG) in a T1W-MRI sequence using six models, including the AlexNet, VGG16, ResNet18, GoogleNet, ResNet50, and MajVot algorithms, and we compared their performances. Each model’s five-trial average (mean) test performance and standard deviation (SD) are provided in Table 6 and compared in Figure 7. Using the suggested MajVot method, the best classification performance for T1W-MRI data was discovered. The test performance of the T1W-MRI data in five rounds is described in Appendix B using the above-mentioned six models. Figure 8 depicts the behavior of each model’s five-fold test accuracy. This demonstrates how the accuracy of CNN models varies significantly across different data folds. At the same time, the MajVot algorithm’s accuracy for T1W-MRI data was discovered to be more stable and consistent across all data folds.

#### 3.1.2. T2W-MRI Data Analysis

**Figure 9:**
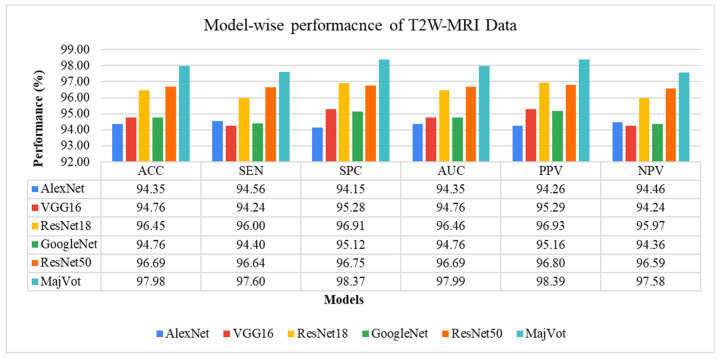
Five-fold test performance of T2W-MRI Data.

**Figure 10:**
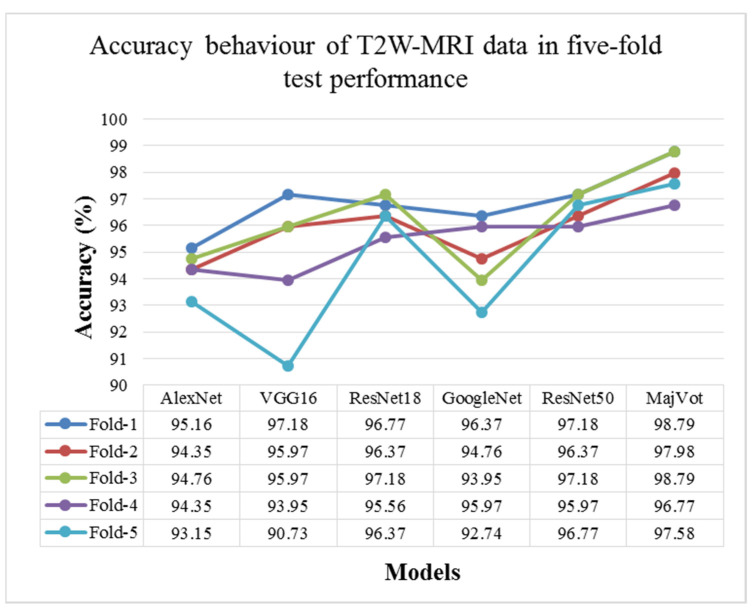
Model-wise accuracy behavior in the five-fold test performance of T2W-MRI data.

In this experiment, six models—AlexNet, VGG16, ResNet18, GoogleNet, ResNet50, and MajVot algorithms were used to analyze the classification performance of LGG against HGG on T2W-MRI sequence data. Table 7 and Figure 9 compare each model’s average test results of five rounds. The proposed MajVot-algorithm was found to classify these data with the highest performance in glioma classification. Appendix C contains the comprehensive findings of five trials of each model. Figure 10 depicts the behavior of each model’s five-fold test accuracy. This demonstrates that CNN models have very uneven accuracy across different data folds. The MajVot algorithm’s accuracy for T2W-MRI data was shown to be consistent and improved across all data folds.

#### 3.1.3. FLAIR-MRI Data Analysis

**Figure 11:**
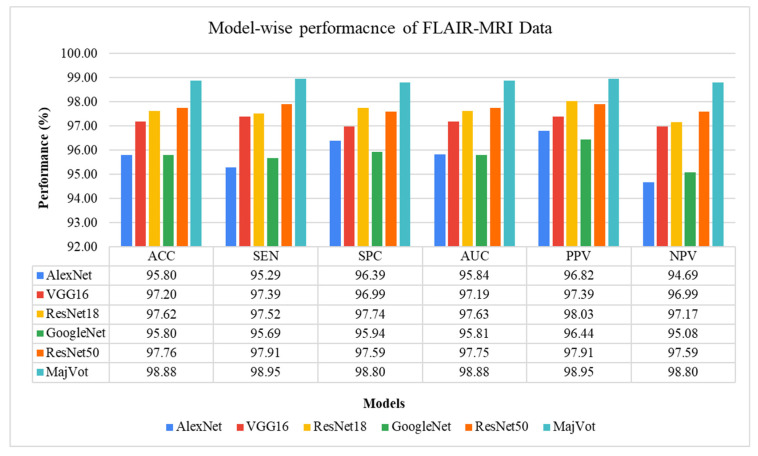
Five-fold test performance of FLAIR-MRI Data.

**Figure 12:**
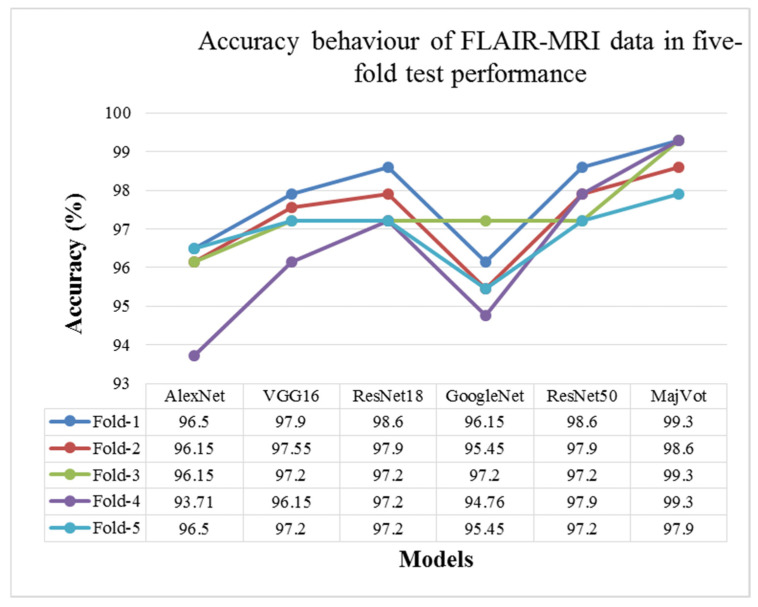
Model-wise accuracy behavior in the five-fold test performance of FLAIR-MRI data.

This analysis tested glioma classification on FLAIR-MRI data using the same six methods and compared them. The average test performance of each model in five rounds is given in Table 8 and compared in Figure 11. The highest classification performance of FLAIR-MRI data was seen using the proposed using MajVot-algorithm. The five trials’ test performance of FLAIR-MRI data using the above-mentioned five models are described in Appendix D. The behavior of the five-fold test accuracy of each model is shown in Figure 12. This indicates that *CNN* models exhibit highly inconsistent performance across different folds of data. At the same time, the accuracy of the MajVot algorithm for FLAIR-MRI data was found to be consistent and better across all folds of data.

### 3.2. Performance Comparison of Three MRI Sequence Datasets

**Figure 13:**
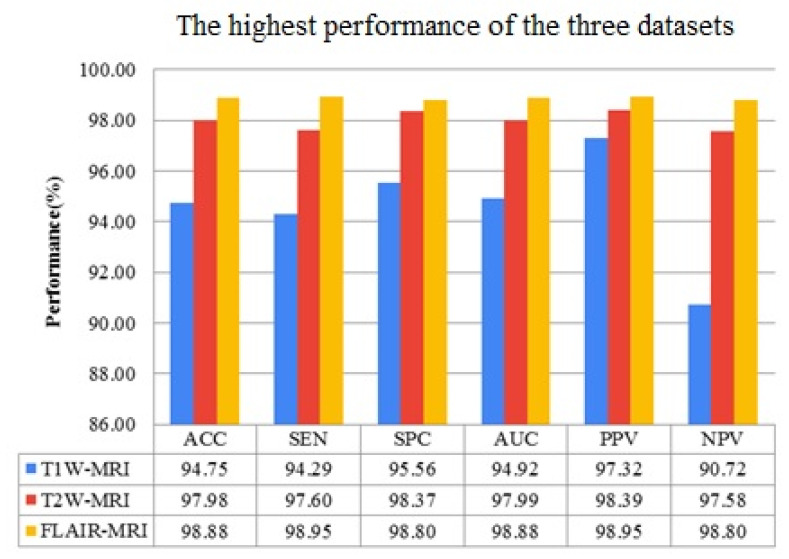
Highest performances of three MRI sequence datasets.

**Figure 14:**
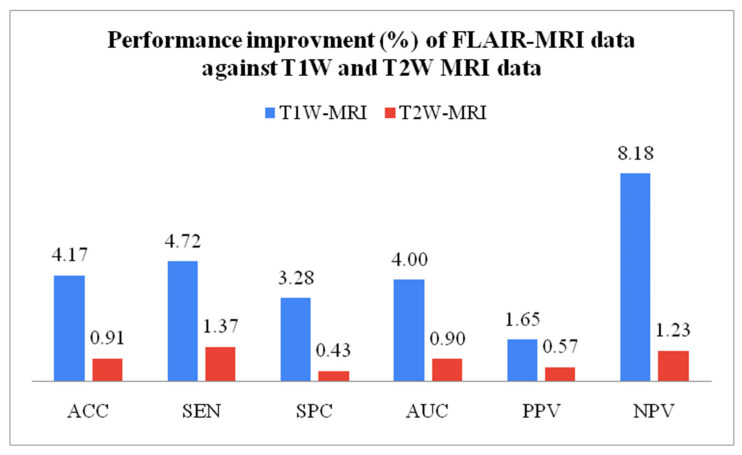
Performance improvement of FLAIR-MRI data against T1W and T2W-MRI data.

The best performance of all datasets is compared in this experimental protocol. Equation (9) mathematically expresses the performance improvement (*IMP*) between two datasets. Variable *a* represents higher data performance, and variable *b* represents lesser data performance. The FLAIR-MRI sequence data outperformed the other two datasets in terms of low-grade versus high-grade classification with *ACC*: 98.88 ± 0.63, *SEN*: 98.95 ± 0.58, *SPC*: 98.80 ± 0.67, *AUC*: 98.88 ± 0.63, *PPV*: 98.95 ± 0.58, *NPV*: 98.80 ± 0.67. This shows 4.17% and 0.91% improvement in accuracy against T1W-MRI and T2W-MRI data, respectively. Table 9 shows the best performance of the three datasets, compared in Figure 13. Similarly, Table 10 compares the percentage of enhanced performance of FLAIR-MRI data against T1W and T2W MRI data and is graphically represented in Figure 14.

(9) $$IMP=\frac{\left(a-b\right)}{a}\times 100$$

### 3.3. Model-Wise Performance Improvement

**Figure 15:**
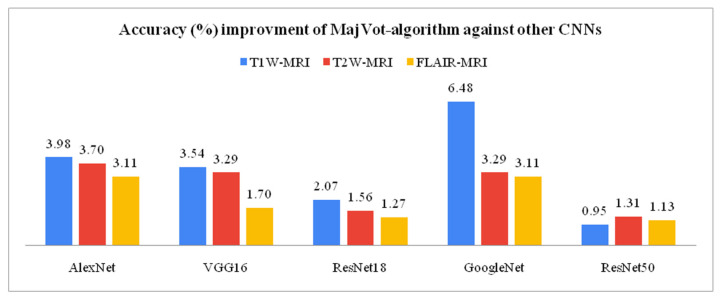
The accuracy improvement of the MajVot algorithm against the other five models on three datasets.

This section compares the model-wise performance of three MRI sequences’ data and analyzes the percentage performance improvement. The performance improvement (*IMP*) between the two models is given by Equation (9). Variable *a* is the highest performance of model-x, and *b* is the lowest performance of model-y. Among six models, the highest LGG versus HGG classification accuracy of T1W-MRI (94.75%), T2W-MRI (97.98%), and FLAIR-MRI data (98.88%) was obtained by the proposed MajVot algorithm. The proposed MajVot algorithm showed 3.98%, 3.54%, 2.07%, 6.48%, and 0.95% accuracy improvement against AlexNet, VGG16, ResNet18, GoogleNet, and ResNet50 models for T1W-MRI data. Similarly, the MajVot algorithm exhibited 3.70%, 3.29%, 1.56%, 3.29%, and 1.31% accuracy improvement against AlexNet, VGG16, ResNet18, GoogleNet, and ResNet50 models for T2W-MRI data. Finally, the MajVot algorithm was depicted at 3.11%, 1.70%, 1.27%, 3.11%, and 1.13% accuracy improvement against AlexNet, VGG16, ResNet18, GoogleNet, and ResNet50 models for FLAIR-MRI data. The maximum accuracy of six models for three datasets is depicted in Table 11, and the percentage improved in the accuracy of the MajVot algorithm against the other five models for three datasets is depicted in Figure 15.

### 3.4. Experimental Protocol 4: Average Performance Analysis of Six Models and Three Datasets

**Figure 16:**
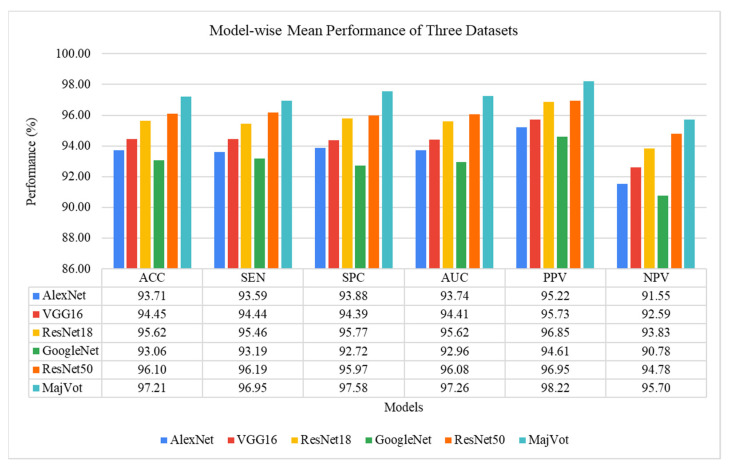
The model-wise average performance of three datasets.

**Figure 17:**
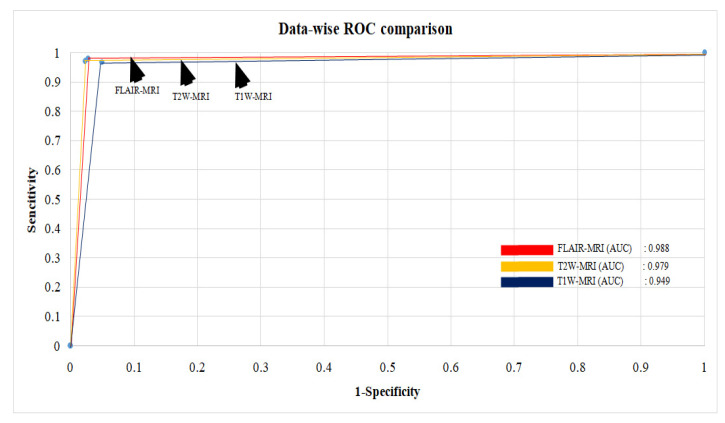
Average ROC between three datasets.

**Figure 18:**
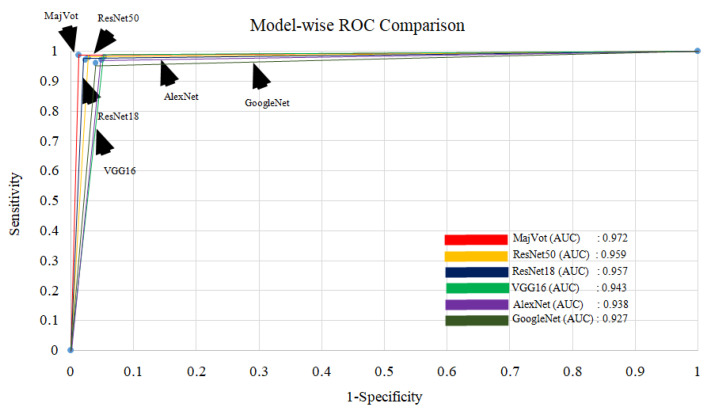
Comparison between ROC of six models.

In this section, model-wise and data-wise average performances are analyzed. The data-wise average $\eta \left(DS_{i}\right)$ is the average performance of six models for the same data and is depicted by Equation (10). Similarly, the mode-wise average $\eta \left(M_{k}\right)$ is the average of the performance of the same model for three datasets and is depicted by Equation (11). The highest average performance of six models among three datasets was observed in FLAIR-MRI data (*ACC*: 97.18 ± 1.10, *SEN*: 97.12 ± 1.27, *SPC*: 97.24 ± 0.94, *AUC*: 97.18 ± 1.08, *PPV*: 97.59 ± 0.83, *NPV*: 96.72 ± 1.42) as depicted in Table 12. Similarly, the highest average performance of the three datasets was obtained using the proposed-MajVot algorithm (*ACC*: 97.21 ± 2.17, *SEN*: 96.95 ± 2.40, *SPC*: 97.58 ± 1.76, *AUC*: 97.26 ± 2.08, *PPV*: 98.22 ± 0.83, *NPV*: 95.70 ± 4.36), given in Table 13 and compared in Figure 16. The average ROC values of three datasets and six models are compared in Figure 17 and Figure 18, respectively.

(10) $$\eta \left(DS_{i}\right)=\overset{6}{\underset{k=1}{\sum }}\frac{\partial_{\left(DS_{i},M_{k}\right)}}{6}$$

(11) $$\eta \left(M_{k}\right)=\sum_{i=1}^{3}\frac{\partial_{\left(DS_{i},M_{k}\right)}}{3}$$

## 4. Discussion

The main objective of this study is to develop an efficient CAD tool for brain tumor grading. Therefore, we aim to find a suitable MRI sequence and efficient algorithm so that the efficiency of the tool can be improved. Thus, in this study, appropriate MRI sequence is searched for from T1W, T2W, and FLAIR, to improve LGG versus HGG classification. Further, to maximize the performance of tumor classification, a majority voting based ensemble algorithm is proposed using five well-known CNN models. A total of 18 experiments were conducted on six models and three datasets. A five-fold cross-validation protocol was used, resulting in a total of 90 cycles of training. Furthermore, four experimental protocols were designed for performance analysis in this experiment. In the first experimental protocol, the data and model-wise performances were demonstrated for six models. The highest performance of three MRI sequence datasets was compared in the second experimental protocol. Model-wise performance improvement was depicted in the third experimental protocol. The average performance of the models and datasets was compared in the fourth experimental protocol. FLAIR-MRI sequence data is found to be the most suitable for LGG versus HGG classification in our experiment, and its highest performance is observed in *ACC*: 98.88 ± 0.63, *SEN*: 98.95 ± 0.58, *SPC:* 98.80 ± 0.67, *AUC:* 98.88 ± 0.63, *PPV:* 98.95 ± 0.58, *NPV*: 98.80 ± 0.67, using a *CNN*-based ensemble algorithm. On the same algorithm, it showed a 4.17% and 0.91% improvement in the accuracy against T1W-MRI and T2W-MRI sequence data. Further, the CNN-based MajVot algorithm produced the highest classification performance for all three MRI sequence datasets. The average performance of the three datasets is as follows *ACC*: 97.21 ± 2.17, *SEN*: 96.95 ± 2.40, *SPC*: 97.58 ± 1.76, *AUC*: 97.26 ± 2.08, *PPV*: 98.22 ± 0.83, *NPV*:95.70 ± 4.36. The proposed MajVot algorithm showed 3.98%, 3.54%, 2.07%, 6.48%, and 0.95% accuracy improvement against AlexNet, VGG16, ResNet18, GoogleNet, and ResNet50 models for T1W-MRI data. Similarly, the MajVot algorithm exhibited 3.70%, 3.29%, 1.56%, 3.29%, and 1.31% accuracy improvement against AlexNet, VGG16, ResNet18, GoogleNet, and ResNet50 models for T2W-MRI data. Finally, the MajVot algorithm was depicted at 3.11%, 1.70%, 1.27%, 3.11%, and 1.13% accuracy improvement against AlexNet, VGG16, ResNet18, GoogleNet, and ResNet50 models for FLAIR-MRI data. So, we can conclude that the FLAIR-MRI sequence is most suitable for brain tumor classification. The proposed DL model-based MajVot algorithm is an excellent method for performance improvement for classification compared to individual deep learning models.

### 4.1. Special Note on Deep Learning Method

These days, deep learning models have become researchers’ first choice in making intelligent machines. Deep learning technology powers the most popular applications of this time, such as voice search [104], AI-based cameras [105], AI games [106], automatic search recommendations [107], etc. The main engine of the DL algorithm is the neural network, which is an imitation of the human brain and is related to its functioning [52,108,109]. However, we must look out for significant challenges of DL systems. Just as the human brain requires a lot of experience to learn and reduce information, DL involves a lot of data to reach the desired level of intelligence [28]. A more significant amount of training data is required to train a successful DL model, but in the absence of sufficient data, it fails to make correct estimates [110]. Therefore, some techniques have been invented to overcome the unavailability of data, such as transfer learning and data augmentation [111,112]. In the medical domain, limited data is always a significant concern [71,113]. Therefore, we have adopted both techniques to address this issue in the proposed work.

The SoftMax function plays an important role for the classification task of DL models [114,115,116]. The SoftMax layer is the key node of the above models, which accepts vectors of real numbers as input and normalizes them into a distribution proportional to the exponential of the input values [117,118]. Sometimes the inputs to the SoftMax may be negative or greater than one, or the sum of the inputs may not be exactly one. The primary objective of the *SoftMax* function is to normalize all outputs to 0–1 and ensure that the total probability of all outcomes is equal to one. The mathematical expression of the *SoftMax* function is shown in Equation (12). Here, *z* is an input vector, the mathematical value of *e* ≈ 2.718, *N* is number of classes, (*z*)*_I_* is the output probability of *ith* class. Let there be three classes {G1, G2, G3} (*N* = 3); their feature vector is *z* = [0.25, 1.23, −0.8], which is evaluated by NN. If this feature vector is given as input to the *SoftMax* function, then the normalized output is corresponding to the class as given by Equation (13). As can be seen in the result, the property of the *SoftMax* function is maintained. First, the probability of each class is between 0–1. Second, the sum of all probabilities is one (0.664 + 0.249 + 0.087 = 1).

(12) $$SoftMax{\left(z\right)}_{i}=\frac{e^{z_{i}}}{\sum_{j=1}^{N}e^{z_{j}}}$$

(13) $$\left[\begin{array}{c} 0.25\\ 1.23\\ -0.8 \end{array}\right]\to \left[SoftMax\right]\to \left[\begin{array}{c} 0.249\\ 0.664\\ 0.087 \end{array}\right]$$

Further, DL is considered an external network where it is excellent at input and output mapping but cannot interpret the proper context. For example, in video games, DL algorithms have a different understanding than humans. In its place, those specific tricks are learned that prevent them from being lost through the trial-and-error method. However, this does not mean that the AI algorithm has the same understanding as humans of the different elements of the game. Since DLs require a lot of data, they also require sufficient computational power to process them. Therefore, data scientists switch to multi-core high-performance GPUs and similar processing units to ensure better efficiency and lower time consumption. These processing units are expensive and consume a lot of power. The DL models are considered ‘black boxes’. Despite knowing about inputs, model parameters, and architecture, there needs to be a reasonable justification for how they draw conclusions [48,76,93]. Transparency is another major issue in areas such as financial trades or medical diagnostics, where users may prefer to understand how a given system makes decisions.

### 4.2. Clinical Applications of Magnetic Resonance Imaging

MRI is free from ionizing radiation and identifies body abnormality better than computed tomography (CT). Further, it offers many alternative imagining sequences through different tissue contrast such as T1-W, T2-W, FLAIR, and proton density in multiple planes such as link, axial, sagittal, and coronal [82]. Because of these features, it is beneficial in early diagnosis, surgical management, post-operative imaging, radiation planning, and response assessment. We employed MRI for brain tumor diagnosis and grade estimation in the present work. Some factors should be noted, which may affect the sequence choice of MRI. The MRI can detect a wide spectrum of central nervous system (CNS) disorders related to the brain, brain stem, and spinal cord. MRI provides excellent contrast sensitivity and is not hindered by the thickness of the skull in this area. Therefore, the sagittal views of MRI are suitable for posterior fossa studies. Low-grade lesions in brain-stem tumors are detected much more clearly in MRI than in CT [108,119]. T1-weighted images are more sensitive to cerebrospinal fluid (CSF) and describe anatomic details of brain tumors with low signal intensity. Sagittal T1-W images are found suitable to detect the abnormality of middle brain structures, particularly the corpus callosum and cerebellum. Anterior visual pathways, schizencephaly, and holoprosencephaly abnormalities can be aptly identified by the coronal T1-W image [10,120]. T2-weighted images are useful for lesion detection, whereas most of the tumor’s lesion and CSF appear in high-signal intensity. FLAIR images are composed of T2-weighted with low-signal CSF and highly sensitive to pathology detection. Therefore, they show most lesions, such as tumors and edema, with higher signal intensity than T2 images. Axial T2-W and coronal FLAIR images can be combined into T2-W images, consequently providing a complementary picture scheme for children under 2 years of age [121].

### 4.3. Strength, Weakness, and Future Extension

So far, most of the similar proposed works have estimated tumor grades through a single MRI sequence dataset. To our knowledge, this is the first exhaustive study of its kind where an appropriate MRI sequence for brain tumor grading has been discovered. Further, the *CNN*-based ensembled approach is a good idea for performance optimization for image classification compared to using single or multiple independent models. In traditional methods of brain tumor classification, skull stripping, region-of-interest definition, feature selection, and feature segmentation are the important steps. Since DL can automatically extract useful features, whole-brain MRI has been used for decision-making without any preprocessing. This method not only saves unnecessary computation effort but also maintains the original characteristics of the tumor, which may be altered after preprocessing. It is a non-invasive procedure that takes less time than a biopsy. Therefore, this method is highly beneficial for detecting brain tumors in the early stage of the disease or can be used as a second opinion of biopsy. However, 130 patient data were used from a single institution. The suggested method’s novelty can be assessed on multi-institutional data or in a real-world scenario with a suitably large training dataset (millions of images). The proposed MajVot algorithm is a five-model-based ensemble method; its performance is still due to test on many models, which could be a valuable topic for future research. The majority vote method in our proposed work considers the opinions of five deep learning models. This approach, however, can be improved by a concept provided by [122], where the retrieved features of each deep learning model may be integrated and the best suitable features for categorization can be chosen. This strategy might improve tumor categorization even further. To further extend future research, more sophisticated ensemble techniques can be designed which can fuse ML-based methods with a multilayered DL solution for automated feature extraction methods.

### 4.4. Benchmarking

Most similar proposed works, such as [69,79,80], have evaluated tumor grade using a single MRI sequence. However, no appropriate MRI sequence for brain tumor classification has yet been established. As a result, this is the first comprehensive study of its kind that compares and investigates MRI sequences suited for brain tumor grading. FLAIR-MRI is found to be a suitable MRI sequence for brain tumor classification. In previous DL-based investigations, two ways of image inputs were used. First, an ROI segmented image was adopted for input to the model by some researchers such as [69,78,80]. On the other hand, whole brain images data were used by researchers such as [79,80]. The DL models can automatically extract useful features. Using this hypothesis, an MRI of the whole brain is sufficient to make decisions without any preprocessing. However, the effect of segmentation method we have already compared in our earlier study [123] and found that segmentation did not have a significant impact on the classification performance. This method not only saves unnecessary computational effort but also preserves the original features of the tumor, which may change after preprocessing. Previously, in all comparative investigations, a single or many models were used; nevertheless, the best performance of a single model was highlighted. In our proposed idea, we used five DL models for training and used a majority vote mechanism to get the opinions of all the models. As a result, the *CNN*-based ensembled strategy outperforms any single independent model in brain tumor classification. The performance of the proposed algorithm and three MRI sequence datasets is compared with existing methods in Table 14.

## 5. Conclusions

The main objective of the proposed study is to develop an efficient MRI-based automated computer-aided tool for brain tumor grading. This method is non-invasive and takes less time than a biopsy. This tool can be used as an alternative to biopsy or as a second option for brain tumor grading. In this study, two main issues are addressed. First, an appropriate MRI sequence was sought for brain tumor classification. Second, the performance of existing models was enhanced using an ensemble algorithm based on majority voting with relevant MRI sequence data. At the same time, the experimental FLAIR-MRI sequence data reported the highest performance using the proposed ensemble algorithm. Additionally, it was noted that different convolutional neural networks gave varied outcomes for their convolutional layers across distinct data folds. The proposed ensemble approach employed the input from five convolutional neural networks, gave consistent results for all data folds in five-fold cross-validation, and outperformed all other individual models in terms of total classification performance. Future ensemble solutions can be designed which fuse ML-based classification paradigms with DL-based feature extractors.

## Figures and Tables

**Table 1:** Sample details of clinically relevant datasets.

Dataset	MRI Sequence	Class Samples		Training Set (80%)		Test Set (20%)		Total Samples
		LGG	HGG	LGG (80%)	HGG (80%)	LGG (20%)	HGG (20%)	
Dataset-1	FLAIR	663	767	530	613	133	154	1430
Dataset-2	T1W	337	560	269	448	68	112	897
Dataset-3	T2W	617	623	493	498	124	125	1240

**Table 2:** Special features of the used pre-trained architecture of CNNs.

Attributes	AlexNet	VGG16	GoogleNet	ResNet18	ResNet50
Layers count	8	16	22	18	50
Input size	227 × 227 × 3	224 × 224 × 3	224 × 224 × 3	224 × 224 × 3	224 × 224 × 3
Model description	Conv: 5, FC: 3	Conv: 13, FC: 3	Conv: 21, FC: 1	Conv: 17, FC: 1	Conv: 49, FC: 1
Special feature	Local Response Normalization, Overlapping Max-Pooling	Object Localization and Image Classification	1 × 1 Convolution Global average pooling Inception module	Skip connections	Skip connections
Top-5 error rate	15.3%	7.3%	6.67%	3.57%	3.57%
Parameters (Million)	60	138	4	11.4	23.9

**Table 3:** Total combinations of experiments (#Experiments = 18).

Models\DataSets	DS_1_	DS_2_	DS_3_
M_1_	(DS_1_, M_1_)	(DS_2_, M_1_)	(DS_3_, M_1_)
M_2_	(DS_1_, M_2_)	(DS_2_, M_2_)	(DS_3_, M_2_)
M_3_	(DS_1_, M_3_)	(DS_2_, M_3_)	(DS_3_, M_3_)
M_4_	(DS_1_, M_4_)	(DS_2_, M_4_)	(DS_3_, M_4_)
M_5_	(DS_1_, M_5_)	(DS_2_, M_5_)	(DS_3_, M_5_)
M_6_	(DS_1_, M_6_)	(DS_2_, M_6_)	(DS_3_, M_6_)

Datasets, DS_1_: T1W-MRI Data, DS_2_:T2W-MRI Data, DS_3_: FLAIR-MRI data, M_1_: AlexNet, M_2_: VGG16, M_3_: ResNet18, M_4_: GoogleNet, M_5_: ResNet50, M_6_: MajVot Algorithm.

**Table 4:** Initial training parameters of CNNs.

Training Parameter	Values
Epochs	100
Batch Size	10
Mean Iterations	5000
Learning Rate	0.0001
Training Protocol	Five-fold cross-validation

**Table 5:** The mathematical expression of mean test performance of five trials of an experiment.

Parameters (Mean of Five Trails)	Mathematical Expression
Mean Accuracy	$\partial_{ACC}=\frac{\sum_{t=1}^{5}ACC_{t}}{5}$
Mean Sensitivity	$\partial_{SEN}=\frac{\sum_{t=1}^{5}SE_{t}}{5}$
Mean Specificity	$\partial_{SPC}=\frac{\sum_{t=1}^{5}SP_{t}}{5}$
Mean Positive Predicted Value	$\partial_{PPV}=\frac{\sum_{t=1}^{5}PPV_{t}}{5}$
Mean Negative Predicted Value	$\partial_{NPV}=\frac{\sum_{t=1}^{5}NPV_{t}}{5}$
Mean Areas Under the Curve	$\partial_{AUC}=\frac{\sum_{t=1}^{5}AUC_{t}}{5}$

**Table 6:** Five-fold test performance of T1W-MRI Data.

Models	$\partial_{ACC}$	$\partial_{SEN}$	$\partial_{SPC}$	$\partial_{AUC}$	$\partial_{PPV}$	$\partial_{NPV}$
	Mean ± SD	Mean ± SD	Mean ± SD	Mean ± SD	Mean ± SD	Mean ± SD
AlexNet	90.98 ± 2.90	90.91 ± 3.01	91.11 ± 3.04	91.01 ± 2.89	94.58 ± 1.88	85.50 ± 4.47
VGG16	91.39 ± 2.54	91.69 ± 2.73	90.89 ± 3.08	91.29 ± 2.57	94.51 ± 1.83	86.56 ± 4.17
ResNet18	92.79 ± 1.22	92.86 ± 1.03	92.67 ± 2.17	92.76 ± 1.37	95.60 ± 1.26	88.35 ± 1.58
GoogleNet	88.61 ± 1.62	89.48 ± 1.41	87.11 ± 2.43	88.30 ± 1.76	92.24 ± 1.42	82.88 ± 2.18
ResNet50	93.85 ± 1.71	94.03 ± 1.48	93.56 ± 2.28	93.79 ± 1.82	96.15 ± 1.36	90.16 ± 2.40
**MajVot Algorithm**	**94.75 ± 0.61**	**94.29 ± 0.54**	**95.56 ± 0.79**	**94.92 ± 0.64**	**97.32 ± 0.47**	**90.72 ± 0.86**

**Table 7:** Five-fold test performance of T2W-MRI Data.

Models	$\partial_{ACC}$	$\partial_{SEN}$	$\partial_{SPC}$	$\partial_{AUC}$	$\partial_{PPV}$	$\partial_{NPV}$
	Mean ± SD	Mean ± SD	Mean ± SD	Mean ± SD	Mean ± SD	Mean ± SD
AlexNet	94.35 ± 0.75	94.56 ± 1.19	94.15 ± 0.68	94.35 ± 0.75	94.26 ± 0.65	94.46 ± 1.15
VGG16	94.76 ± 2.53	94.24 ± 3.01	95.28 ± 2.10	94.76 ± 2.53	95.29 ± 2.15	94.24 ± 2.95
ResNet18	96.45 ± 0.60	96.00 ± 0.57	96.91 ± 0.68	96.46 ± 0.60	96.93 ± 0.67	95.97 ± 0.57
GoogleNet	94.76 ± 1.48	94.40 ± 1.70	95.12 ± 1.29	94.76 ± 1.48	95.16 ± 1.30	94.36 ± 1.68
ResNet50	96.69 ± 0.53	96.64 ± 0.67	96.75 ± 0.57	96.69 ± 0.53	96.80 ± 0.56	96.59 ± 0.67
**MajVot Algorithm**	**97.98 ± 0.86**	**97.60 ± 0.98**	**98.37 ± 0.81**	**97.99 ± 0.85**	**98.39 ± 0.81**	**97.58 ± 0.97**

**Table 8:** Five-fold test performance of FLAIR-MRI Data.

Models	$\partial_{ACC}$	$\partial_{SEN}$	$\partial_{SPC}$	$\partial_{AUC}$	$\partial_{PPV}$	$\partial_{NPV}$
	Mean ± SD	Mean ± SD	Mean ± SD	Mean ± SD	Mean ± SD	Mean ± SD
AlexNet	95.80 ± 1.19	95.29 ± 1.26	96.39 ± 1.45	95.84 ± 1.19	96.82 ± 1.26	94.69 ± 1.40
VGG16	97.20 ± 0.65	97.39 ± 0.46	96.99 ± 0.92	97.19 ± 0.67	97.39 ± 0.79	96.99 ± 0.54
ResNet18	97.62 ± 0.63	97.52 ± 0.85	97.74 ± 0.53	97.63 ± 0.61	98.03 ± 0.46	97.17 ± 0.95
GoogleNet	95.80 ± 0.93	95.69 ± 0.75	95.94 ± 1.14	95.81 ± 0.94	96.44 ± 0.99	95.08 ± 0.86
ResNet50	97.76 ± 0.59	97.91 ± 0.55	97.59 ± 0.63	97.75 ± 0.59	97.91 ± 0.55	97.59 ± 0.63
**MajVot Algorithm**	**98.88 ± 0.63**	**98.95 ± 0.58**	**98.80 ± 0.67**	**98.88 ± 0.63**	**98.95 ± 0.58**	**98.80 ± 0.67**

**Table 9:** Highest classification performance of three MRI sequence data.

DataSet	*ACC*	*SEN*	*SPC*	*AUC*	*PPV*	*NPV*
	Mean ± SD	Mean ± SD	Mean ± SD	Mean ± SD	Mean ± SD	Mean ± SD
T1W-MRI	94.75 ± 0.61	94.29 ± 0.54	95.56 ± 0.79	94.92 ± 0.64	97.32 ± 0.47	90.72 ± 0.86
T2W-MRI	97.98 ± 0.86	97.60 ± 0.98	98.37 ± 0.81	97.99 ± 0.85	98.39 ± 0.81	97.58 ± 0.97
**FLAIR-MRI**	**98.88 ± 0.63**	**98.95 ± 0.58**	**98.80 ± 0.67**	**98.88 ± 0.63**	**98.95 ± 0.58**	**98.80 ± 0.67**

**Table 10:** Performance improvement of FLAIR data against T1W and T2W data.

IMP (%) of FLAIR-MRI Data	*ACC*	*SEN*	*SPC*	*AUC*	*PPV*	*NPV*
T1W-MRI Data	4.17%	4.72	3.28	4.00	1.65	8.18
T2W-MRI Data	0.91%	1.37	0.43	0.90	0.57	1.23

**Table 11:** The maximum accuracy of six models on three datasets.

Model	T1W-MRI	T2W-MRI	FLAIR-MRI
AlexNet	90.98	94.35	95.80
VGG16	91.39	94.76	97.20
ResNet18	92.79	96.45	97.62
GoogleNet	88.61	94.76	95.80
ResNet50	93.85	96.69	97.76
**MajVot-Algorithm**	**94.75**	**97.98**	**98.88**

**Table 12:** Average performance of six models for each dataset.

Dataset	*ACC*	*SEN*	*SPC*	*AUC*	*PPV*	*NPV*
T1W-MRI	92.06 ± 2.02	92.21 ± 1.70	91.81 ± 2.62	92.01 ± 2.14	95.07 ± 1.58	87.36 ± 2.72
T2W-MRI	95.83 ± 1.31	95.57 ± 1.27	96.10 ± 1.40	95.84 ± 1.31	96.14 ± 1.37	95.54 ± 1.27
**FLAIR-MRI**	**97.18 ± 1.10**	**97.12 ± 1.27**	**97.24 ± 0.94**	**97.18 ± 1.08**	**97.59 ± 0.83**	**96.72 ± 1.42**

**Table 13:** The model-wise average performance of three datasets.

Model	*ACC*	*SEN*	*SPC*	*AUC*	*PPV*	*NPV*
AlexNet	93.71 ± 2.47	93.59 ± 2.35	93.88 ± 2.65	93.74 ± 2.47	95.22 ± 1.39	91.55 ± 5.24
VGG16	94.45 ± 2.92	94.44 ± 2.85	94.39 ± 3.15	94.41 ± 2.97	95.73 ± 1.49	92.59 ± 5.41
ResNet18	95.62 ± 2.52	95.46 ± 2.38	95.77 ± 2.72	95.62 ± 2.54	96.85 ± 1.22	93.83 ± 4.78
GoogleNet	93.06 ± 3.89	93.19 ± 3.28	92.72 ± 4.88	92.96 ± 4.07	94.61 ± 2.15	90.78 ± 6.84
ResNet50	96.10 ± 2.02	96.19 ± 1.98	95.97 ± 2.13	96.08 ± 2.05	96.95 ± 0.89	94.78 ± 4.03
**MajVot Algorithm**	**97.21 ± 2.17**	**96.95 ± 2.40**	**97.58 ± 1.76**	**97.26 ± 2.08**	**98.22 ± 0.83**	**95.70 ± 4.36**

**Table 14:** The comparison of the proposed method with existing methods.

SN	Reference	Data Source	Class	MRI-Sequence	Preprocessing	Model	CV	Highest Accuracy (%)
1	Yang et al. [69]	TCIA (REMBRANDT)	2	T1W	ROI	AlexNet and GoogleNet	K5	94.5%
2	Khawaldeh et al. [79]	TCIA (REMBRANDT)	3	FLAIR	Whole image	Modified AlexNet	NA	91.16%
3	Alies et al. [78]	NA	2	T1W-c T2W FLAIR	ROI/Whole image	ANN	NA	88.3%
4	Anaraki et al. [80]	TCIA (REMBRANDT) and Others	3/4	T1W	ROI Segmented	Proposed CNN	NA	94.2%
5	Swati et al. [81]	Figshar Data	3	T1W	Whole image	VGG19	K5	94.82%
6	Badža et al. [82]	Tianjin Medical University, China	3	T1W	Whole image	Proposed CNN	K10	96.56%
**7**	**Our method**	**TCIA (REMBRANDT)**	**2**	**T1W** **T2W** **FLAIR**	**Whole image**	**MajVot (AlexNet, VGG16,** **ResNet18,** **GoogleNet,** **ResNet50)**	**K5**	**FLAIR-MRI (98.88%)** **T2W-MRI (97.98%)** **T1W-MRI (94.75%)**

TCIA: The Cancer Imaging Archive, REMBRANDT: Repository of Molecular Brain Neoplasia Data, CV: Cross-validation, K5: Five-fold, K10: Ten-Fold, ROI: Region of interest.

**Figure A5:**
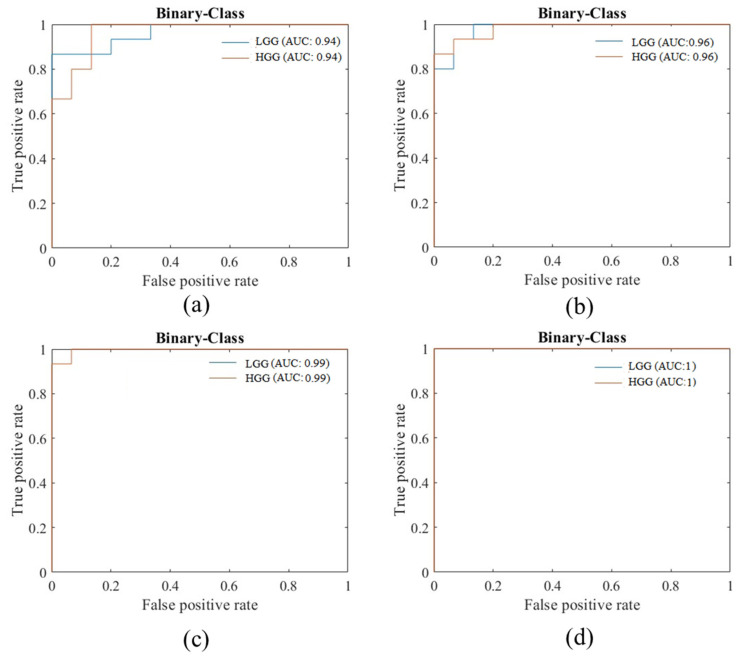
Sample ROC curves of four rounds of training, (**a**) ROC for AUC: 0.94, (**b**) ROC for AUC: 0.96, (**c**) ROC for AUC: 0.99, (**d**) ROC for AUC: 1.

## Data Availability

The brain tumor data are downloaded from the public data repository “The Cancer Imaging Archive (TCIA)”. This is a repository of molecular brain neoplasia data (REMBRANDT). The data can be downloaded from the following URL link: https://wiki.cancerimagingarchive.net/display/Public/REMBRANDT/ (accessed on 20 January 2022).

## Appendix A

**Table A1:** Comparison of the earlier proposed works.

S.No.	Reference	Year	Summary	Novelty
1	Gupta et al. [78]	2022	A combined model with InceptionResNetV2 and Random Forest Tree was proposed for brain tumor classification. The model achieved 99% and 98% accuracy for the suggested tumor classification and detection models, respectively.	Two-model fusion
2	Haq E et al. [73]	2022	CNN and ML are combined to improve the accuracy of tumor segmentation and classification. The suggested technique attained the maximum classification accuracy of 98.3% between gliomas, meningiomas, and pituitary tumors.	Extracted features of CNN and ML models are fused
3	Srinivas et al. [74]	2022	Three CNN models are used in transfer learning mode for brain tumor classification: VGG16, ResNet50, and Inception-v3. The VGG16 has the best accuracy of 96% in classifying tumors as benign or malignant.	Compare three CNN models in transfer learning mode for brain tumor classification
4	Almalki et al. [75]	2022	Classified tumors using a linear machine learning classifiers (MLCs) model and a DL model. The proposed CNN with several layers (19, 22, and 25) is used to train the multiple MLCs in transfer learning to extract deep features. The accuracy of the CNN-SVM fused model was higher than that of previous MLC models. The fused model provided the highest accuracy (98%).	CNN-SVM mode fused for classification
5	Kibriya et al. [76]	2022	Suggested a new deep feature fusion-based multiclass brain tumor classification framework. Deep CNN features were extracted from transfer learning architectures such as AlexNet, GoogleNet, and ResNet18, and fused to create a single feature vector. SVM and KNN models are used as a classifier on this feature vector. The fused feature vector outperforms the individual vectors and system, achieving 99.7% highest accuracy.	Features of three CNNs are combined in a single feature vector
6	Gurunathan et al. [77]	2022	Suggested a CNN Deep net classifier for detecting brain tumors and classifying them into low and high grades. The suggested technique claims segmentation and classification accuracy of 99.4% and 99.5%, respectively.	Proposed a CNN Deep net classifier

**Table A2:** Sample distribution of five-fold cross-validation.

Data	MRI (FLAIR)		MRI (T1W)		MRI (T2W)	
#Fold	Training	Test	Training	Test	Training	Test
Fold 1	1143	287	717	180	991	249
Fold 2	1143	287	717	180	991	249
Fold 3	1143	287	717	180	991	249
Fold 4	1143	287	717	180	991	249
Fold 5	1143	287	717	180	991	249

## Appendix B

**Table A3:** Test results of five-folds of experiment (AlexNet, T1W).

Experiment	Round#	*ACC*	*SEN*	*SPC*	*AUC*	*PPV*	*NPV*	*ITR*	*TT* (Minutes)
(AlexNet, T1W)	1	90.16	90.91	88.89	89.90	93.33	85.11	9900	46.00
	2	95.08	94.81	95.56	95.18	97.33	91.49	9900	45.00
	3	92.62	92.86	92.22	92.54	95.33	88.30	9900	47.00
	4	89.34	88.31	91.11	89.71	94.44	82.00	9900	55.00
	5	87.70	87.66	87.78	87.72	92.47	80.61	9900	54.00
	**MEAN**	**90.98**	**90.91**	**91.11**	**91.01**	**94.58**	**85.50**	**9900**	**49.40**
	**SD**	**2.90**	**3.01**	**3.04**	**2.89**	**1.88**	**4.47**	**0.00**	**4.72**

*ACC*: Accuracy, *SEN*: Sensitivity, *SPC*: Specificity, *AUC*: Area under the curve, *PPV*: Positive Predictive Value, *NPV*: Negative Predictive Value, *ITR*: Iterations, *TT*: Training Time.

**Table A4:** Test results of five-folds of experiment (VGG16, T1W).

Experiment	Round#	*ACC*	*SEN*	*SPC*	*AUC*	*PPV*	*NPV*	*ITR*	*TT* (Minutes)
(ResNet18, T1W)	1	95.49	96.10	94.44	95.27	96.73	93.41	9900	315.00
	2	90.98	90.91	91.11	91.01	94.59	85.42	9900	313.00
	3	91.39	90.26	93.33	91.80	95.86	84.85	9900	341.00
	4	90.57	92.21	87.78	89.99	92.81	86.81	9900	394.00
	5	88.52	88.96	87.78	88.37	92.57	82.29	9900	354.00
	**MEAN**	**91.39**	**91.69**	**90.89**	**91.29**	**94.51**	**86.56**	**9900**	**343.40**
	**SD**	**2.54**	**2.73**	**3.08**	**2.57**	**1.83**	**4.17**	**0.00**	**33.20**

**Table A5:** Test results of five-folds of experiment (ResNet18, T1W).

Experiment	Round#	*ACC*	*SEN*	*SPC*	*AUC*	*PPV*	*NPV*	*ITR*	*TT* (Minutes)
(ResNet18, T1W)	1	91.39	91.56	91.11	91.33	94.63	86.32	9900	146.00
	2	94.67	94.16	95.56	94.86	97.32	90.53	9900	92.00
	3	92.62	92.21	93.33	92.77	95.95	87.50	9900	117.00
	4	93.03	92.86	93.33	93.10	95.97	88.42	9900	86.00
	5	92.21	93.51	90.00	91.75	94.12	89.01	9900	112.00
	**MEAN**	**92.79**	**92.86**	**92.67**	**92.76**	**95.60**	**88.35**	**9900**	**110.60**
	**SD**	**1.22**	**1.03**	**2.17**	**1.37**	**1.26**	**1.58**	**0.00**	**23.70**

**Table A6:** Test results of five-folds of experiment (GoogleNet, T1W).

Experiment	Round#	*ACC*	*SEN*	*SPC*	*AUC*	*PPV*	*NPV*	*ITR*	*TT* (Minutes)
(GoogleNet, T1W)	1	90.16	90.26	90.00	90.13	93.92	84.38	9900	54.00
	2	89.75	90.91	87.78	89.34	92.72	84.95	9900	98.00
	3	89.34	90.26	87.78	89.02	92.67	84.04	9900	112.00
	4	87.30	87.66	86.67	87.16	91.84	80.41	9900	141.00
	5	86.48	88.31	83.33	85.82	90.07	80.65	9900	121.00
	**MEAN**	**88.61**	**89.48**	**87.11**	**88.30**	**92.24**	**82.88**	**9900**	**105.20**
	**SD**	**1.62**	**1.41**	**2.43**	**1.76**	**1.42**	**2.18**	**0.00**	**32.60**

**Table A7:** Test results of five-folds of experiment (ResNet50, T1W).

Experiment	Round#	*ACC*	*SEN*	*SPC*	*AUC*	*PPV*	*NPV*	*ITR*	*TT* (Minutes)
(ResNet50, T1W)	1	95.49	95.45	95.56	95.51	97.35	92.47	9900	230.00
	2	95.08	95.45	94.44	94.95	96.71	92.39	9900	245.00
	3	94.67	94.16	95.56	94.86	97.32	90.53	9900	246.00
	4	92.21	92.86	91.11	91.98	94.70	88.17	9900	346.00
	5	91.80	92.21	91.11	91.66	94.67	87.23	9900	256.00
	**MEAN**	**93.85**	**94.03**	**93.56**	**93.79**	**96.15**	**90.16**	**9900.00**	**264.60**
	**SD**	**1.71**	**1.48**	**2.28**	**1.82**	**1.36**	**2.40**	**0.00**	**46.44**

**Table A8:** Test results of five-folds of experiment (MajVot, T1W).

Experiment	Round#	*ACC*	*SEN*	*SPC*	*AUC*	*PPV*	*NPV*
(MajVot, T1W)	1	95.08	94.81	95.56	95.18	97.33	91.49
	2	94.67	94.16	95.56	94.86	97.32	90.53
	3	95.49	94.81	96.67	95.74	97.99	91.58
	4	94.67	94.16	95.56	94.86	97.32	90.53
	5	93.85	93.51	94.44	93.98	96.64	89.47
	**MEAN**	**94.75**	**94.29**	**95.56**	**94.92**	**97.32**	**90.72**
	**SD**	**0.61**	**0.54**	**0.79**	**0.64**	**0.47**	**0.86**

## Appendix C

**Table A9:** Test results of five-folds of the experiment (AlexNet, T2W).

Experiment	Round#	*ACC*	*SEN*	*SPC*	*AUC*	*PPV*	*NPV*	*ITR*	*TT* (Minutes)
(AlexNet, T2W)	1	95.16	95.20	95.12	95.16	95.20	95.12	9900	46.00
	2	94.35	94.40	94.31	94.35	94.40	94.31	9900	45.00
	3	94.76	96.00	93.50	94.75	93.75	95.83	9900	47.00
	4	94.35	94.40	94.31	94.35	94.40	94.31	9900	55.00
	5	93.15	92.80	93.50	93.15	93.55	92.74	9900	54.00
	**MEAN**	**94.35**	**94.56**	**94.15**	**94.35**	**94.26**	**94.46**	**9900.00**	**49.40**
	**SD**	**0.75**	**1.19**	**0.68**	**0.75**	**0.65**	**1.15**	**0.00**	**4.72**

**Table A10:** Test results of five-folds of the experiment (VGG16, T2W).

Experiment	Round#	*ACC*	*SEN*	*SPC*	*AUC*	*PPV*	*NPV*	*ITR*	*TT* (Minutes)
(ResNet18, T2W)	1	97.18	96.80	97.56	97.18	97.58	96.77	9900	315.00
	2	95.97	96.00	95.93	95.97	96.00	95.93	9900	313.00
	3	95.97	96.00	95.93	95.97	96.00	95.93	9900	341.00
	4	93.95	92.80	95.12	93.96	95.08	92.86	9900	394.00
	5	90.73	89.60	91.87	90.73	91.80	89.68	9900	354.00
	**MEAN**	**94.76**	**94.24**	**95.28**	**94.76**	**95.29**	**94.24**	**9900.00**	**343.40**
	**SD**	**2.53**	**3.01**	**2.10**	**2.53**	**2.15**	**2.95**	**0.00**	**33.20**

**Table A11:** Test results of five-folds of the experiment (ResNet18, T2W).

	Round#	*ACC*	*SEN*	*SPC*	*AUC*	*PPV*	*NPV*	*ITR*	*TT* (Minutes)
(ResNet18, T2W)	1	96.77	96.00	97.56	96.78	97.56	96.00	9900	146.00
	2	96.37	96.00	96.75	96.37	96.77	95.97	9900	92.00
	3	97.18	96.80	97.56	97.18	97.58	96.77	9900	117.00
	4	95.56	95.20	95.93	95.57	95.97	95.16	9900	86.00
	5	96.37	96.00	96.75	96.37	96.77	95.97	9900	112.00
	**MEAN**	**96.45**	**96.00**	**96.91**	**96.46**	**96.93**	**95.97**	**9900.00**	**110.60**
	**SD**	**0.60**	**0.57**	**0.68**	**0.60**	**0.67**	**0.57**	**0.00**	**23.70**

**Table A12:** Test results of five-folds of the experiment (GoogleNet, T2W).

Experiment	Round#	*ACC*	*SEN*	*SPC*	*AUC*	*PPV*	*NPV*	*ITR*	*TT* (Minutes)
(GoogleNet, T2W)	1	96.37	96.00	96.75	96.37	96.77	95.97	9900	315.00
	2	94.76	94.40	95.12	94.76	95.16	94.35	9900	313.00
	3	93.95	93.60	94.31	93.95	94.35	93.55	9900	341.00
	4	95.97	96.00	95.93	95.97	96.00	95.93	9900	394.00
	5	92.74	92.00	93.50	92.75	93.50	92.00	9900	354.00
	**MEAN**	**94.76**	**94.40**	**95.12**	**94.76**	**95.16**	**94.36**	**9900.00**	**343.40**
	**SD**	**1.48**	**1.70**	**1.29**	**1.48**	**1.30**	**1.68**	**0.00**	**33.20**

**Table A13:** Test results of five-folds of the experiment (ResNet50, T2W).

Experiment	Round#	*ACC*	*SEN*	*SPC*	*AUC*	*PPV*	*NPV*	*ITR*	*TT* (Minutes)
(ResNet50, T2W)	1	97.18	96.80	97.56	97.18	97.58	96.77	9900	315.00
	2	96.37	96.00	96.75	96.37	96.77	95.97	9900	313.00
	3	97.18	97.60	96.75	97.17	96.83	97.54	9900	341.00
	4	95.97	96.00	95.93	95.97	96.00	95.93	9900	394.00
	5	96.77	96.80	96.75	96.77	96.80	96.75	9900	354.00
	**MEAN**	**96.69**	**96.64**	**96.75**	**96.69**	**96.80**	**96.59**	**9900.00**	**343.40**
	**SD**	**0.53**	**0.67**	**0.57**	**0.53**	**0.56**	**0.67**	**0.00**	**33.20**

**Table A14:** Test results of five-folds of the experiment (MajVot, T2W).

Experiment	Round#	*ACC*	*SEN*	*SPC*	*AUC*	*PPV*	*NPV*
(MajVot, T2W)	1	98.79	98.40	99.19	98.79	99.19	98.39
	2	97.98	97.60	98.37	97.99	98.39	97.58
	3	98.79	98.40	99.19	98.79	99.19	98.39
	4	96.77	96.00	97.56	96.78	97.56	96.00
	5	97.58	97.60	97.56	97.58	97.60	97.56
	**MEAN**	**97.98**	**97.60**	**98.37**	**97.99**	**98.39**	**97.58**
	**SD**	**0.86**	**0.98**	**0.81**	**0.85**	**0.81**	**0.97**

## Appendix D

**Table A15:** Test results of five-folds of the experiment (AlexNet, FLAIR).

Experiment	Round#	*ACC*	*SEN*	*SPC*	*AUC*	*PPV*	*NPV*	*ITR*	*TT* (Minutes)
(AlexNet, FLAIR)	1	96.50	96.73	96.24	96.49	96.73	96.24	9900	46.00
	2	96.15	94.77	97.74	96.26	97.97	94.20	9900	45.00
	3	96.15	95.42	96.99	96.21	97.33	94.85	9900	47.00
	4	93.71	93.46	93.98	93.72	94.70	92.59	9900	55.00
	5	96.50	96.08	96.99	96.54	97.35	95.56	9900	54.00
	**MEAN**	**95.80**	**95.29**	**96.39**	**95.84**	**96.82**	**94.69**	**9900.00**	**49.40**
	**SD**	**1.19**	**1.26**	**1.45**	**1.19**	**1.26**	**1.40**	**0.00**	**4.72**

**Table A16:** Test results of five-folds of the experiment (VGG16, FLAIR).

Experiment	Round#	*ACC*	*SEN*	*SPC*	*AUC*	*PPV*	*NPV*	*ITR*	*TT* (Minutes)
(ResNet18, FLAIR)	1	97.90	98.04	97.74	97.89	98.04	97.74	9900	315.00
	2	97.55	97.39	97.74	97.56	98.03	97.01	9900	313.00
	3	97.20	97.39	96.99	97.19	97.39	96.99	9900	341.00
	4	96.15	96.73	95.49	96.11	96.10	96.21	9900	394.00
	5	97.20	97.39	96.99	97.19	97.39	96.99	9900	354.00
	**MEAN**	**97.20**	**97.39**	**96.99**	**97.19**	**97.39**	**96.99**	**9900.00**	**343.40**
	**SD**	**0.65**	**0.46**	**0.92**	**0.67**	**0.79**	**0.54**	**0.00**	**33.20**

**Table A17:** Test results of five-folds of the experiment (ResNet18, FLAIR).

Experiment	Round#	*ACC*	*SEN*	*SPC*	*AUC*	*PPV*	*NPV*	*ITR*	*TT* (Minutes)
(ResNet18, FLAIR)	1	98.60	98.69	98.50	98.59	98.69	98.50	9900	146.00
	2	97.90	98.04	97.74	97.89	98.04	97.74	9900	92.00
	3	97.20	97.39	96.99	97.19	97.39	96.99	9900	117.00
	4	97.20	96.73	97.74	97.24	98.01	96.30	9900	86.00
	5	97.20	96.73	97.74	97.24	98.01	96.30	9900	112.00
	**MEAN**	**97.62**	**97.52**	**97.74**	**97.63**	**98.03**	**97.17**	**9900.00**	**110.60**
	**SD**	**0.63**	**0.85**	**0.53**	**0.61**	**0.46**	**0.95**	**0.00**	**23.70**

**Table A18:** Test results of five-folds of the experiment (GoogleNet, FLAIR).

Experiment	Round#	*ACC*	*SEN*	*SPC*	*AUC*	*PPV*	*NPV*	*ITR*	*TT* (Minutes)
(GoogleNet, FLAIR)	1	96.15	96.08	96.24	96.16	96.71	95.52	9900	146.00
	2	95.45	95.42	95.49	95.46	96.05	94.78	9900	92.00
	3	97.20	96.73	97.74	97.24	98.01	96.30	9900	117.00
	4	94.76	94.77	94.74	94.75	95.39	94.03	9900	86.00
	5	95.45	95.42	95.49	95.46	96.05	94.78	9900	112.00
	**MEAN**	**95.80**	**95.69**	**95.94**	**95.81**	**96.44**	**95.08**	**9900.00**	**110.60**
	**SD**	**0.93**	**0.75**	**1.14**	**0.94**	**0.99**	**0.86**	**0.00**	**23.70**

**Table A19:** Test results of five-folds of the experiment (ResNet50, FLAIR).

Experiment	Round#	*ACC*	*SEN*	*SPC*	*AUC*	*PPV*	*NPV*	*ITR*	*TT* (Minutes)
(ResNet50, FLAIR)	1	98.60	98.69	98.50	98.59	98.69	98.50	9900	146.00
	2	97.90	98.04	97.74	97.89	98.04	97.74	9900	92.00
	3	97.20	97.39	96.99	97.19	97.39	96.99	9900	117.00
	4	97.90	98.04	97.74	97.89	98.04	97.74	9900	86.00
	5	97.20	97.39	96.99	97.19	97.39	96.99	9900	112.00
	**MEAN**	**97.76**	**97.91**	**97.59**	**97.75**	**97.91**	**97.59**	**9900.00**	**110.60**
	**SD**	**0.59**	**0.55**	**0.63**	**0.59**	**0.55**	**0.63**	**0.00**	**23.70**

**Table A20:** Test results of five-folds of the experiment (MajVot, FLAIR).

Experiment	Round#	*ACC*	*SEN*	*SPC*	*AUC*	*PPV*	*NPV*
(MajVot, FLAIR)	1	99.30	99.35	99.25	99.30	99.35	99.25
	2	98.60	98.69	98.50	98.59	98.69	98.50
	3	99.30	99.35	99.25	99.30	99.35	99.25
	4	99.30	99.35	99.25	99.30	99.35	99.25
	5	97.90	98.04	97.74	97.89	98.04	97.74
	**MEAN**	**98.88**	**98.95**	**98.80**	**98.88**	**98.95**	**98.80**
	**SD**	**0.63**	**0.58**	**0.67**	**0.63**	**0.58**	**0.67**
